# The Role of Neuropeptide Y in Dental Pulp: Balancing Neurogenic Inflammation and Pain Modulation

**DOI:** 10.1111/iej.70192

**Published:** 2026-06-05

**Authors:** Javier Caviedes‐Bucheli, Esteban Ulate, Hugo‐Roberto Munoz, Nestor Ríos‐Osorio

**Affiliations:** ^1^ Centro de Investigaciones Odontológicas Pontificia Universidad Javeriana Bogotá Colombia; ^2^ Facultad de Odontología, Postgrado de Endodoncia Universidad de Costa Rica San José Costa Rica; ^3^ Endodontics Department Universidad de San Carlos de Guatemala Guatemala City Guatemala; ^4^ Endodontic Research Department, School of Dentistry, CES University Medellin Colombia; ^5^ Faculty of Dentistry Institución Universitaria Visión de las Américas Medellín Colombia

**Keywords:** dental pulp, neurogenic inflammation, neuroimmune responses, neuropeptide Y, nociception/pain modulation, pulpal biology, vascular regulation

## Abstract

**Background:**

Dental pulp is a densely innervated, low‐compliance tissue in which neurogenic inflammation can rapidly escalate into oedema, raised intrapulpal pressure, microvascular compromise, and pain. While Substance P (SP) and Calcitonin gene‐related peptide (CGRP) are well‐established drivers of pulp vasodilation, the Neuroopeptide Y (NPY) endogenous counter‐regulatory mechanism remain comparatively under‐explored.

**Objectives:**

To critically synthesise current evidence on NPY in dental pulp biology, focusing on its roles in neurogenic inflammation, neuroimmune modulation, vascular control and nociceptive signalling, and to evaluate whether NPY release represents a physiologically meaningful intrinsic analgesic mechanism during restorative‐procedure–related pulpal stress and dentine hypersensitivity.

**Methods:**

A PRISMA‐guided literature search was conducted in PubMed, Web of Science and Scopus from inception to February 2026, supplemented by grey literature and reference screening. Experimental (in vitro/in vivo/ex vivo), human and animal tissue‐based studies, and relevant reviews reporting molecular, cellular, or functional data on NPY and its receptors (particularly Y1/Y2) in the context of pulp inflammation and pain were included. Study selection and data extraction were performed independently by two reviewers.

**Results:**

Ninety‐two studies met inclusion criteria for narrative synthesis. Across models, NPY emerged as a master homeostatic regulator acting via Gi/Go‐coupled receptors to oppose sensory neuropeptide–driven excitation. Y1 signalling, expressed in vascular smooth muscle and multiple pulp cell populations, mediates vasoconstriction and exerts antinociceptive effects partly through inhibition of TRPV1‐expressing nociceptors and suppression of CGRP release. In other tissues, Y2 signalling functions predominantly presynaptically to limit SP/CGRP spillover, establishing a negative‐feedback ‘brake’ on neurogenic escalation, suggesting it may have a role in dental pulp as well. Stage‐dependent changes in NPY/Y1 expression in pulp of carious teeth indicate an early protective programme that may fail as tissue damage and denervation advance. Mechanistic and clinical data converge on a model in which pulp outcomes reflect the balance between excitatory (SP/CGRP) and NPY‐mediated inhibitory tone.

**Conclusion:**

NPY signalling constitutes a potential counter‐regulatory axis integrating vascular control, neuroimmune responses, and pain modulation in dental pulp. NPY may function as an intrinsic analgesic mechanism that limits procedure‐ and caries‐associated neurogenic inflammation and dentine hypersensitivity.

## 1 Introduction

The dental pulp is a highly specialised mesenchymal tissue with dense sensory, sympathetic and parasympathetic innervation. Sensory afferent fibres arise from the trigeminal ganglion, sympathetic fibres from the cervical sympathetic ganglion, and parasympathetic input is thought to originate predominantly from the pterygopalatine (sphenopalatine) ganglion (Caviedes‐Bucheli and Munoz 2025). This complex neurovascular architecture underpins neurogenic inflammation, mediated by the release of neuropeptides with potent vasoregulatory and immunomodulatory effects (Caviedes‐Bucheli, Muñoz, et al. 2008).

Confined within a rigid, low‐compliance dentinal chamber, the dental pulp is highly susceptible to inflammatory stimuli, which rapidly increase intrapulpal pressure, compress the microvasculature, and induce local hypoxia (Heyeraas and Kvinnsland 1992). Pulpal vessels exhibit unusually thin walls compared with systemic vasculature, rendering them highly responsive to vasoactive neuropeptides. In this confined environment, neuropeptide‐induced vasodilation may exacerbate tissue oedema, further elevate intrapulpal pressure, and aggravate microvascular compromise (Zhan et al. 2021). Within this specialised microenvironment, neuropeptides function not only as neurotransmitters, but also as growth factors, hormonal mediators and key modulators of immune responses (Lundy and Linden 2004). The most extensively studied neuropeptides in dental pulp biology include Calcitonin gene‐related peptide (CGRP), Substance P (SP), Neurokinin A (NKA), Vasoactive intestinal peptide (VIP) and Neuropeptide Y (NPY) (Caviedes‐Bucheli et al. 2006).

Pain perception in the dental pulp is primarily mediated by orthodromic conduction, the classical nociceptive pathway in which action potentials generated at peripheral terminals propagate towards the central nervous system via the trigeminal ganglion, leading to conscious pain perception (Henry and Hargreaves 2007). This process is initiated when mechanical, thermal, or chemical stimuli activate channels such as TRPV, TRPA1 and Piezo, allowing Na^+^ and Ca^2+^ influx that generates a depolarising receptor potential; once threshold is reached, voltage‐gated sodium channels (Nav1.7, Nav1.8) open, producing an action potential (Caterina and Julius 2001; Renton et al. 2003; Yu et al. 2005; Szczot et al. 2021). Neuropeptide release is triggered by this depolarization and subsequent Ca^2+^ influx at nerve terminals (Kawashima and Okiji 2024). In addition to orthodromic transmission, action potentials can propagate antidromically through collateral branches via an axon reflex, whereby local depolarising currents bring adjacent membrane segments to threshold, sequentially opening Na^+^ channels and allowing the signal to spread within the peripheral arborisation; this process does not require activation from the trigeminal ganglion, although the same impulse may simultaneously travel centrally (Lai et al. 2004; Chung et al. 2013; Sorkin et al. 2018). The pseudo‐unipolar architecture of trigeminal neurones enables this dual propagation, allowing a single neurone to mediate both nociception and local effector responses. Pseudo‐unipolar neurones are primary sensory neurones in which a single axonal process bifurcates into peripheral and central branches, allowing direct transmission of sensory signals to the central nervous system while also enabling antidromic conduction between peripheral terminals, a mechanism fundamental to neurogenic inflammation in the dental pulp (Lin and Chen 2018).

Under physiological conditions, mild stimuli predominantly activate Aδ fibres, while autonomic fibres release VIP and NPY to regulate vascular tone (Kim 1990; El‐Karim et al. 2003). In contrast, noxious stimuli preferentially activate C fibres, leading to release of CGRP and substance P, which induce neurogenic inflammation characterised by vasodilation, increased permeability, elevated intrapulpal pressure, and immune cell recruitment (Caviedes‐Bucheli et al. 2006; Caviedes‐Bucheli, Muñoz, et al. 2008). These neuropeptides further sensitise nociceptors via cAMP/PKA signalling, lowering activation thresholds and amplifying action potential generation, thereby enhancing both peripheral inflammation and orthodromic pain transmission. The resulting inflammatory environment contributes to pulpal pain and is modulated by sympathetic release of NPY, which promotes vasoconstriction and counterbalances CGRP/SP mediated vasodilation (Shiau 2012; Lee et al. 2019; Ronan et al. 2024).

Neurogenic inflammation in the dental pulp can be elicited by a broad range of noxious stimuli, including thermal, electrical, bacterial and mechanical inputs, which activate peptidergic sensory fibres and trigger the release of neuropeptides into the pulpal microenvironment (Caviedes‐Bucheli, Muñoz, et al. 2008; Caviedes‐Bucheli et al. 2021). This response has been characterised as a hallmark of pulpal injury, whereby stimulation of pulpal nociceptive fibres induces the release of somatosensory neuropeptides such as CGRP, SP and NKA, promoting vasodilation, plasma extravasation, and immune cell recruitment (Caviedes‐Bucheli et al. 2006; Ahmed and Mahdee 2025). Mechanical stimuli are particularly relevant in clinical practice, as restorative procedures can induce odontoblastic micro‐injury, thermal and frictional stress, and direct mechanical irritation of the pulp tissue, thereby activating sensory fibres within the confined, low‐compliance pulpal environment (Bryniarska‐Kubiak et al. 2024). The magnitude of the pulpal response is modulated by residual dentine thickness, operative technique and pressure, instrument characteristics, and the biocompatibility of restorative materials (Mjör and Ferrari 2002; Caviedes‐Bucheli et al. 2005; Mjör 2009). While healthy pulps generally tolerate minor insults, sustained or intense stimulation may drive progression from reversible inflammation to irreversible pulpitis and eventual pulp necrosis (Mjör and Odont 2001; European Society of Endodontology 2019).

Postganglionic sympathetic fibres are unmyelinated and reach the dental pulp either alongside arterioles through the apical foramen or in association with sensory fibres via the trigeminal ganglion (Byers and Närhi 1999; Olgart 1996). Within the pulp, these fibres are predominantly distributed around blood vessels, forming a perivascular plexus and terminating on the smooth muscle cells of the tunica media (Byers and Närhi 1999; Olgart 1996). Through this arrangement, sympathetic innervation modulates vascular tone by regulating smooth muscle contraction, thereby controlling vessel diameter and pulpal blood flow (Olgart 1996). In healthy pulp tissue, sympathetic fibres are mainly localised within the pulp core and cell‐rich zone, with limited presence in the cell‐free zone, odontoblastic layer, or dentine (Wakisaka 1990; Uddman et al. 1998). However, under pathological conditions such as caries, these fibres exhibit sprouting into the odontoblastic layer and are less confined to perivascular regions (Shimeno et al. 2008). This redistribution suggests that sympathetic innervation may play a role in vascular regulation, and since its effect opposes the vasodilatory effects of CGRP and SP, it could be argued that it counterbalances their effects. This model of reciprocal regulation has been described before regarding DPSC (dental pulp stem cells) and sympathetic fibres. The sympathetic nervous system inhibits the proliferation and migration of DPSCs, and CGRP has been shown to indirectly support dentinogenesis via angiogenesis (Zhan et al. 2024). CGRP and Sonic Hedgehog (SHH) have also been shown to promote DPSC differentiation (Moore et al. 2022). Therefore, the inhibition of sympathetic fibres on DPSCs counterbalances the effects of other neuropeptides and is achieved through metabolic reprogramming, shifting DPSC metabolism from oxidative phosphorylation to anaerobic glycolysis. This effect might be leveraged to maintain these stem cells' quiescent state and preserve their regenerative capabilities (Yang et al. 2023; Luo et al. 2026).

Autonomic fibres release neuropeptides such as NPY from sympathetic fibres and VIP from parasympathetic fibres, contributing to compensatory homeostatic mechanisms that limit excessive inflammatory activity (El Karim, Lamey, Linden, et al. 2006; Rethnam et al. 2010). NPY emerges as a pivotal regulator, functioning not only as a potent vasoconstrictor but also as a neuroimmune modulator capable of influencing nociceptive signalling (El Karim et al. 2008; Gibbs and Hargreaves 2008). Within the dental pulp, NPY is predominantly localised around blood vessels, within the odontoblastic layer, and in the predentine zone, a distribution that strategically supports the regulation of vascular tone and sensory transduction (Caviedes‐Bucheli, Muñoz, et al. 2008).

VIP is a parasympathetic neuropeptide that exerts vasodilatory and immunomodulatory effects through cAMP‐dependent signalling pathways, contributing to the regulation of pulpal blood flow and maintenance of tissue homeostasis (El Karim, Lamey, Ardill, et al. 2006). In addition to its vascular effects, VIP has also been shown to have immunomodulatory properties. Its presence has been demonstrated in immune and inflammatory cells, and VIP modulates the immune response by inhibiting pro‐inflammatory cytokines and up‐regulating anti‐inflammatory cytokines, such as IL‐10, IL‐4 and IL‐9 (Pozo et al. 2000). VIP expression is increased in dental pulps from carious teeth compared with non‐carious adult human teeth. This neuropeptide acts as a neuroprotective molecule that counteracts pro‐inflammatory effects by activating Th2 and Treg cells, both anti‐inflammatory lymphocytes (de Campos Soriani Azevedo et al. 2019). Its role as a trigger of protective responses in other chronic inflammation models, such as multiple sclerosis and arthritis (Tan and Waschek 2011), supports a model in the dental pulp in which neuropeptides govern the balance between the host pro‐inflammatory immune response and the counteracting anti‐inflammatory and reparative responses.

NPY‐mediated vasoconstriction reduces pulpal blood flow and intrapulpal pressure, inhibits acetylcholine‐ and SP‐induced vasodilation, and potentiates the post‐synaptic effects of other vasoconstrictors such as noradrenaline (Lundy and Linden 2004; Caviedes‐Bucheli, Muñoz, et al. 2008). Through these actions, NPY modulates the vasodilatory effects of SP and CGRP, limiting pulpal volume expansion and preventing antidromic fluid movement into dentinal tubules (Kim 1990; El Karim, Lamey, Linden, et al. 2006; El Karim et al. 2008; Rethnam et al. 2010). Consequently, NPY functions as a key defensive regulator of pulpal inflammation. NPY mediates its biological effects via a family of G‐protein‐coupled receptors, including Y1, Y2, Y4, Y5 and Y6, with the Y1 receptor most prominently implicated in vasoconstriction, suppression of neurogenic inflammation, and modulation of nociceptive signalling (Larhammar and Salaneck 2004). Activation of the NPY Y1 receptor has been proposed as an intrinsic analgesic mechanism, capable of limiting excessive sensory neuropeptide release and attenuating nociceptive transmission during pulpal inflammatory processes (El Karim et al. 2008; Gibbs and Hargreaves 2008). In addition, the presence of NPY1 receptors in fibroblasts, undifferentiated cells, and odontoblasts has been proposed to link NPY signalling with pulp mineralisation processes as part of a broader defensive response (Killough et al. 2010; Rethnam et al. 2010). This underscores the role of NPY not merely as a compensatory vasoconstrictor (Olgart 1996), but as an intrinsic analgesic pathway, with potential therapeutic implications for modulating pain perception during dental interventions (Gibbs and Hargreaves 2008).

Despite the extensive body of literature emphasising the roles of CGRP and substance P in pulpal neurogenic inflammation, a comprehensive understanding of pulpal neurobiology remains incomplete without consideration of the multifaceted regulatory functions of NPY. This regulatory and counterbalance model has been observed in other neuroinflammatory processes such as dry eye disease, where NPY levels in tears are reduced and inversely correlated with the severity of dry eye disease due to the anti‐inflammatory properties of NPY (Lambiase et al. 2011). Conversely, reduced CGRP levels were inversely correlated with NPY. This evidence suggests a model in which NPY may counterbalance the effects of sensory neuropeptides in the dental pulp, as dry eye disease is also governed by neuropeptides (Hwang et al. 2021).

Accordingly, this review aims to critically synthesise current evidence on the role of neuropeptide Y in dental pulp biology, with particular emphasis on its regulation of vascular tone, nociceptive signalling, and immune responses. Specifically, it seeks to evaluate whether NPY release constitutes a physiologically relevant intrinsic analgesic mechanism during neurogenic inflammation and dentine hypersensitivity associated with restorative dental procedures, in order to verify if NPY could act as a central modulator of pulpal neuroinflammation and pain.

## 2 Materials and Methods

### 2.1 Focused Question

The inclusion criteria and search strategy were developed using this focused question:

Does NPY release constitute a meaningful intrinsic analgesic mechanism during neurogenic inflammation and dentine hypersensitivity associated with restorative dental procedures?

### 2.2 Inclusion Criteria

Experimental research studies (in vitro, in vivo, ex vivo), human and animal tissue‐based studies, and review articles assessing the molecular mechanisms, cellular signalling pathways, or physiological effects of NPY in dental pulp, neurogenic inflammation or dentine hypersensitivity were included. Studies were eligible if they provided experimental, observational, qualitative, or quantitative data on the expression, regulation, or functional role of NPY, its receptors (particularly Y1), or associated neuropeptides (SP, CGRP, VIP) in modulating pulpal inflammation, vascular responses, or nociceptive signalling. Mechanistic insights linking NPY activity to potential intrinsic analgesic effects in the context of restorative procedures or exposed dentine were considered essential.

### 2.3 Exclusion Criteria

Case reports, editorials, expert opinions, letters to the editor, research unrelated to dental pulp neurobiology or neurogenic inflammation, and studies lacking molecular or cellular data on NPY or other neuropeptides were excluded. Studies focused primarily on non‐neurogenic mechanisms of dentine hypersensitivity or unrelated odontogenic pathologies were not considered.

### 2.4 Information Sources

A comprehensive search strategy was developed using Descriptors in Health Sciences (DeCS), Medical Subject Headings (MeSH), Emtree terms and relevant text words, tailored to each database. The literature search was conducted across MEDLINE (via PubMed), Web of Science, and Scopus from inception to February 2026, without language restrictions. To maximise coverage, additional sources were explored, including reference lists of key articles, thesis repositories, conference proceedings, Open Grey, Google Scholar and ClinicalTrials.gov.

### 2.5 Search Strategy

#### 2.5.1 MEDLINE/PubMed

(“Neuropeptide Y”[Mesh] OR “Neuropeptide Y” OR NPY OR “neuropeptides”) AND (“Dental Pulp”[Mesh] OR “dental pulp” OR pulp OR “pulp tissue”) AND (Inflammation OR “neurogenic inflammation” OR “pulpal inflammation” OR “dentinal hypersensitivity” OR “dentine hypersensitivity” OR nociception OR “pain” OR “pain modulation” OR “pain perception”).

#### 2.5.2 Scopus

TITLE‐ABS‐KEY (“Neuropeptide Y” OR NPY OR neuropeptides) AND TITLE‐ABS‐KEY (“dental pulp” OR pulp OR “pulp tissue”) AND TITLE‐ABS‐KEY (inflammation OR “neurogenic inflammation” OR “pulpal inflammation” OR “dentinal hypersensitivity” OR “dentine hypersensitivity” OR nociception OR pain OR “pain modulation” OR “pain perception”).

#### 2.5.3 Web of Science

TS = (“Neuropeptide Y” OR NPY OR neuropeptides) AND TS = (“dental pulp” OR pulp OR “pulp tissue”) AND TS = (inflammation OR “neurogenic inflammation” OR “pulpal inflammation” OR “dentinal hypersensitivity” OR “dentine hypersensitivity” OR nociception OR pain OR “pain modulation” OR “pain perception”).

This search strategy was designed to capture all relevant studies investigating NPY in dental pulp physiology and pathology, including its role in pain modulation, vascular responses, and immune activity, in both human and animal models.

### 2.6 Study Selection and Data Extraction (Data Collection)

The literature search and study selection were conducted in accordance with the PRISMA guidelines (Page et al. 2021). Two independent reviewers screened records based on titles and abstracts, followed by a full‐text assessment against the pre‐defined inclusion and exclusion criteria. Discrepancies were resolved through discussion, with a third reviewer consulted as necessary to ensure methodological rigour and impartiality. Key data were extracted, including study type, experimental model, NPY expression or activity, receptor involvement, neurogenic inflammation parameters, and evidence of analgesic effects or modulation of vascular/nociceptive responses.

## 3 Results

**FIGURE 1:**
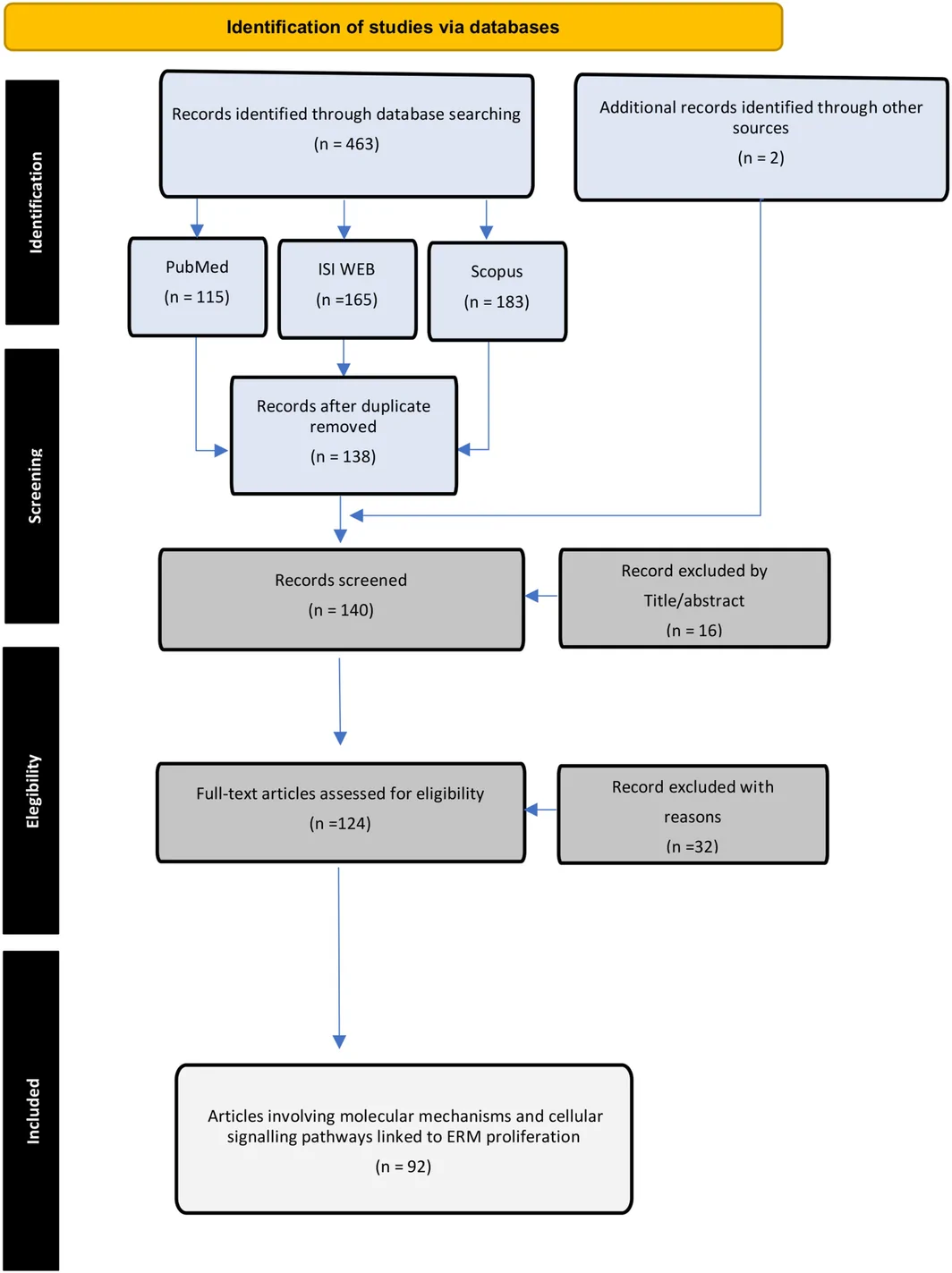
Flowchart of literature search and selection based on PRISMA guidelines.

Initially, 138 articles were retrieved based on the described search strategy, with an additional two records identified through other sources. A preliminary screening of titles and abstracts was performed (*n* = 140), after which 124 full‐text articles were assessed for eligibility according to predetermined inclusion and exclusion criteria. Thirty‐two articles were excluded for specific reasons, leaving 92 studies that met the inclusion criteria for narrative analysis and synthesis (Figure 1).

This review provides a comprehensive, in‐depth, and integrated narrative assessment of the selected literature, focusing on the cellular and molecular mechanisms underlying neurogenic inflammation in the dental pulp, the regulation of vascular tone and nociceptive signalling, and the modulatory role of neuropeptide Y. Given the complexity of pulp neurobiology and the heterogeneity of experimental approaches, a full systematic review was not feasible; instead, this work synthesises mechanistic evidence from molecular, cellular, and clinical studies to elucidate the role of NPY as an intrinsic modulator of pulpal inflammation and pain.

## 4 Literature Review

### 4.1 Revisiting Pulpal Neurovascular Biology: Foundations for Understanding Neuroimmune Modulation

The dental pulp is a highly specialised connective tissue confined within rigid dentinal walls, where minimal inflammatory or nociceptive stimuli can produce disproportionate biological effects due to its dense neurovascular organisation (Kim 1990; Heyeraas and Kvinnsland 1992; Lundy and Linden 2004; Zhan et al. 2021). Before addressing the regulatory role of NPY, it is essential to outline the neurovascular and neuroimmune mechanisms that govern pulpal homeostasis and contribute to nociceptive hypersensitivity.

Pulpal perfusion is maintained by a dense terminal capillary network intimately juxtaposed with sensory nerve fibres (Aδ and C fibres), facilitating bidirectional neurovascular crosstalk. This architectural arrangement, whilst efficient for homeostasis, renders the pulp critically vulnerable due to its low‐compliance environment. Upon exposure to noxious stimuli—including thermal fluctuations, hydrodynamic perturbations, or restorative trauma—sensory afferents release vasoactive neuropeptides (SP and CGRP), triggering plasma extravasation and elevated intrapulpal pressure (Heyeraas and Berggreen 1999; Donaldson 2006; Berggreen et al. 2020). This pressure increment compromises venous return and induces focal hypoxic stress, activating molecular mediators including HIF‐1α and the NLRP3 inflammasome (Jiang et al. 2022; Wang et al. 2024), thereby accelerating the transition from reversible inflammation to irreversible tissue necrosis (Kawashima and Okiji 2024).

Consequently, the rigid dentinal enclosure transforms physiological inflammatory responses into oedema, ischaemia, and progressive pulpal degeneration (Pohl et al. 2024). This constrained physiological milieu predisposes the dental pulp to disproportionately amplified biological responses to otherwise modest stimuli, underpinning its characteristic exaggerated nociception and rapid escalation of inflammatory signalling. Limited interstitial compliance, dependence on terminal arteriolar supply, and dense perivascular innervation collectively create a system in which even subtle vascular dysregulation exerts a profound influence on pain perception and inflammatory dynamics (Heyeraas and Berggreen 1999; Donaldson 2006; Berggreen et al. 2020).

The autonomic nervous system, encompassing both sympathetic and parasympathetic divisions, plays a fundamental role in regulating tissue homeostasis beyond classical haemodynamic control (El Karim, Lamey, Ardill, et al. 2006; Caviedes‐Bucheli, Muñoz, et al. 2008; Rethnam et al. 2010). A principal function of sympathetic activity is the maintenance of vascular tone and blood pressure through arteriolar vasoconstriction. However, sympathetic vasomotor control appears to be of limited relevance for circulatory homeostasis of the jaws, acting instead at a predominantly local level (Olgart 1996).

Importantly, sympathetic innervation also exerts trophic and angiogenic effects, with NPY emerging as a key mediator of vascular growth and tissue repair, underscoring the relevance of sympathetic nerves during pulpal development and regenerative responses (Zukowska‐Grojec et al. 1998; Rethnam et al. 2010).

### 4.2 Neurogenic Inflammation as a Bidirectional Reflex Arc Rather Than a Local Event

Pulp inflammation is now recognised as an integrated neuro‐immune reflex rather than a purely local immune response. Sensory afferents, particularly C fibres, actively propagate inflammation through the antidromic release of vasoactive neuropeptides such as substance P and CGRP at peripheral terminals, rather than serving merely as passive nociceptive conduits (Caviedes‐Bucheli, Muñoz, et al. 2008). Concurrently, orthodromic conduction transmits nociceptive signals to central pathways, while peripheral neuropeptide release elicits rapid vascular modulation and immune cell recruitment independently of classical leukocyte activation, a process collectively termed neurogenic inflammation (Byers et al. 2003; Lundy and Linden 2004). Such bidirectional signalling endows the pulp with exquisite sensitivity, enabling immediate responses to tissue insult. Furthermore, it establishes a self‐reinforcing feed‐forward loop where early neuropeptide release amplifies vasodilation, plasma extravasation, and mast‐cell degranulation (Caviedes‐Bucheli, Muñoz, et al. 2008; Korkmaz et al. 2023). This cycle is critically relevant in the pathogenesis of dentine hypersensitivity and the pulpal response to routine restorative procedures. In these scenarios, mechanical and thermal triggers activate a specific subset of Transient Receptor Potential (TRP) channels—notably TRPV1, TRPA1, and TRPM8—expressed on both odontoblasts and sensory nerve terminals (Hossain et al. 2019). The activation of these mechanosensitive and thermosensitive channels facilitates the rapid release of pro‐inflammatory mediators, thereby sustaining the neurogenic inflammatory state and lowering the nociceptive threshold (Aminoshariae and Kulild 2021). Consequently, the dental pulp's unique reliance on TRP‐mediated transduction transforms transient clinical stimuli into a self‐perpetuating cycle of neurovascular sensitization.

### 4.3 Classical Neuropeptides (SP, CGRP, VIP) Drive Vascular Dysregulation and Hypersensitivity

Within the intricate network of neurogenic signalling in the pulp‐dentine complex, SP and CGRP emerge as the most extensively characterised effectors. SP predominantly mediates plasma extravasation and mast‐cell degranulation via NK1 receptors, whereas CGRP ‐one of the most potent endogenous vasodilators in craniofacial tissues‐regulates pulpal perfusion and lowers nociceptive thresholds (Lundy and Linden 2004; Caviedes‐Bucheli, Muñoz, et al. 2008). In dentine hypersensitivity, antidromic signalling drives fluid movement into the dentinal tubules, where Aδ fibres trigger the initial hydrodynamic response. Subsequent release of SP and CGRP from peptidergic C fibres further amplifies mechanosensitivity and sustains peripheral sensitisation (Caviedes‐Bucheli, Muñoz, et al. 2008). This self‐reinforcing cycle underlies the persistence of hypersensitivity following deep caries removal or restorative interventions. Furthermore VIP, primarily of parasympathetic origin, acts in concert with sensory neuropeptides to intensify neurogenic oedema and prolong vascular reactivity (Rodd et al. 1998). Collectively, these mediators establish a pro‐inflammatory milieu that predisposes the pulp to the transition from transient physiological responses to sustained pathological hypersensitivity (Galler et al. 2021).

### 4.4 Setting the Stage for NPY: The Need for an Endogenous Counter‐Regulatory Mechanism

Given the excitatory effects of SP and CGRP, the dental pulp requires intrinsic mechanisms to restrain neurogenic inflammation and limit nociceptive amplification. While the pro‐inflammatory roles of SP and CGRP are well documented, counter‐regulatory neuropeptides remain under‐explored, leaving a key question: how does the dental pulp prevent excessive neurogenic inflammatory responses and the development of persistent hypersensitivity?

Within this highly reactive neurovascular milieu, NPY emerges as a key counter‐regulatory mediator with vasoconstrictive, anti‐inflammatory and antinociceptive properties (Wakisaka et al. 1996; El Karim, Lamey, Linden, et al. 2006; El Karim et al. 2008; Rethnam et al. 2010). Unlike the broadly distributed SP and CGRP, NPY is predominantly localised to sympathetic perivascular fibres, enabling tight modulation of pulpal blood flow and opposition to SP/CGRP‐driven vasodilation (Silva et al. 2002; Caviedes‐Bucheli, Muñoz, et al. 2008; Caviedes‐Bucheli et al. 2021). NPY expression after dental trauma was highest in avulsion cases but SP expression showed higher concentrations in healthy pulps and in pulps following traumatic injuries (Neiva et al. 2015). This difference could reflect the number of sensory fibres innervating the dental pulp compared to the number of sympathetic fibres. Compared to VIP, the expression of NPY in the dental pulp has been shown to be two times bigger, reflecting the relative importance of sympathetic fibres in the dental pulp compared to parasympathetic fibres (Caviedes‐Bucheli et al. 2006). Quantitative data on neuropeptide Y (NPY) concentrations in human dental pulp are limited; however, studies indicate that NPY is present at low picomolar levels and increases in response to inflammatory stimuli and traumatic injury (Caviedes‐Bucheli, Muñoz, et al. 2008; Killough et al. 2010; Neiva et al. 2015). These findings collectively suggest that NPY is dynamically regulated during pulpal inflammation, albeit at lower levels than sensory neuropeptides (Uddman et al. 1984; Caviedes‐Bucheli et al. 2006; El Karim, Lamey, Linden, et al. 2006).

In addition, activation of Y1 and Y2 receptors confers direct antinociceptive effects by limiting peripheral sensitisation and suppressing sensory neuropeptide release (Nie and Taylor 2025). Thus, pulpal pathology reflects the balance between excitatory sensory neuropeptides and NPY‐mediated counter‐regulation. Adequate NPY activity restrains neurogenic inflammation and preserves pulp vitality, whereas insufficient control promotes hypersensitivity and irreversible inflammatory damage. Conversely, excessive sympathetic vasoconstriction may impair pulpal perfusion. Collectively, NPY emerges as a central homeostatic modulator of pulpal neurovascular and neuroimmune balance (Caviedes‐Bucheli and Munoz 2025). NPY‐containing nerve fibres and its receptor (Y1) are distributed in the apical and coronal parts of the pulp, and previous studies have shown the co‐existence of NPY and CGRP nerve fibres in dental pulp tissue of rats, suggesting a possible modulation between these neuropeptides. Moreover, to counterbalance the vasodilatory effects of CGPR and SP, it has been shown that NPY‐mediated vasoconstriction is long‐lasting and resistant to α‐adrenoceptor antagonists. In peripheral blood vessels, NPY may act on vascular tone by direct constriction, potentiation of noradrenaline‐induced contraction, or inhibition of presynaptic noradrenaline release (Uddman et al. 1998).

Sympathetic innervation plays a dynamic role in the pulpal response to dental caries, characterised by increased expression of NPY and its Y1 receptor in carious compared with non‐carious teeth. This expression is maximal in mild to moderate lesions and declines markedly in advanced caries, reflecting a stage‐dependent regulatory function. During early and intermediate disease, NPY modulates nociception, immune activity, angiogenesis and reparative dentinogenesis, whereas in advanced lesions, tissue necrosis and loss of sympathetic fibres abolish this control, permitting unrestrained neurogenic and inflammatory responses (El Karim et al. 2008). In contrast, although parasympathetic innervation does not appear to play a major role in the regulation of pulpal blood flow compared with the sympathetic nervous system (Olgart 1996), a modest increase in VIP expression has been reported in mild and moderate stages of dental caries relative to healthy pulps (El Karim, Lamey, Ardill, et al. 2006). This parasympathetic contribution likely represents a compensatory mechanism that counterbalances NPY‐mediated sympathetic vasoconstriction, thereby fine‐tuning pulpal microcirculation and helping to preserve tissue viability during the early phases of cariogenic injury (Caviedes‐Bucheli, Muñoz, et al. 2008; Caviedes‐Bucheli and Munoz 2025).

**FIGURE 2:**
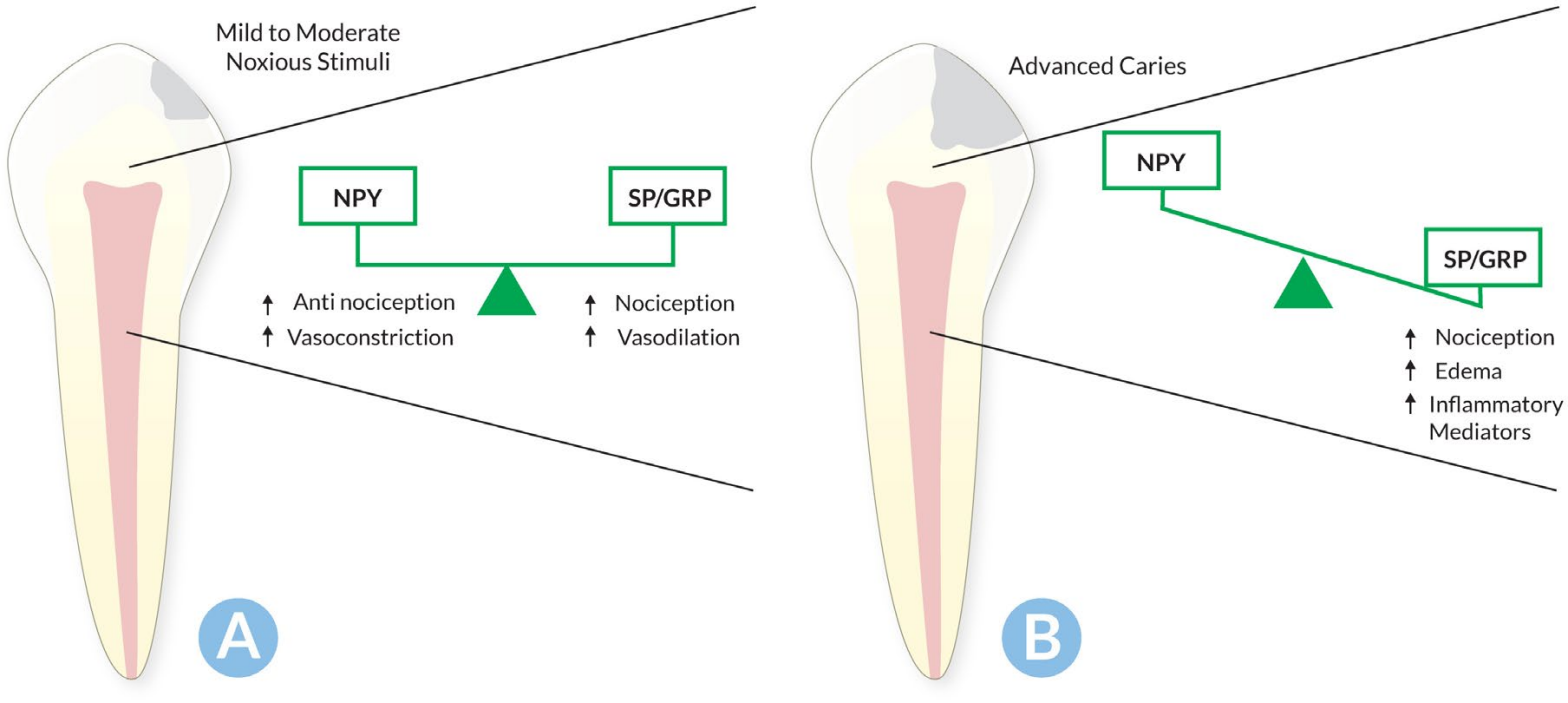
Sympathetic–sensory neuropeptide imbalance as a determinant of pulpal disease progression. The interplay between sensory neuropeptides (SP/CGRP) and NPY governs the transition from reversible to irreversible pulpitis during mild to moderate caries. Expression of NPY and its receptors peaks (see A) and declines sharply in advanced caries (see B). Sufficient NPY‐mediated regulation restrains neurogenic signalling and counterbalances SP/CGRP‐mediated pro‐inflammatory effects. If sympathetic fibres suffer extensive damage due to advanced noxious stimuli, sensory neuropeptides‐mediated effects continue unchecked (see B).

In carious teeth, NPY is predominantly localised to free nerve endings within the dental pulp and to fibres extending towards the odontoblastic layer and predentine, often independent of vascular structures, suggesting a direct role in tissue repair during early and moderate caries (Zukowska‐Grojec et al. 1998; El Karim, Lamey, Linden, et al. 2006). This reparative function is likely mediated via Y1 receptor expression on pulpal fibroblasts, which act as primary targets of NPY signalling (Killough et al. 2010). Functionally, NPY stimulates fibroblast‐driven tertiary dentinogenesis through upregulation of bone morphogenetic protein‐2 (BMP‐2), enhancing odontoblast differentiation and secretory activity, while concurrently promoting angiogenesis by inducing endothelial cell migration, proliferation, and capillary formation (El Karim et al. 2009). Given the phenotypic plasticity of pulpal fibroblasts and their capacity to differentiate into dentine‐forming cells, NPY‐mediated neurovascular–fibroblast crosstalk emerges as a central mechanism in pulpal repair and regeneration following cariogenic injury (Hargreaves et al. 2003) (Figure 2).

Building upon the neurovascular and neuroimmune framework described above, the functional impact of NPY in the pulp is inherently dependent on the receptor architecture that mediates its actions. All NPY receptor subtypes (Y1, Y2, Y4, Y5 and Y6) signal predominantly through Gi/Go proteins, which inhibit adenylate cyclase and consequently reduce intracellular cAMP levels (Larhammar and Salaneck 2004; Tanaka et al. 2021). This unified inhibitory signalling stands in stark contrast to the excitatory pathways mediated by SP via Gq‐coupled NK1 receptors and CGRP via Gs‐coupled CALCRL/RAMP1 complexes (Teodoro et al. 2013; Russell et al. 2014). These excitatory pathways promote vasodilation, mast‐cell degranulation, and neuronal sensitisation, whereas the NPY receptor family collectively establishes an intrinsic anti‐excitatory axis that serves as a physiological brake on neurogenic inflammation (El Karim, Lamey, Linden, et al. 2006; El Karim et al. 2008; Killough et al. 2010; Rethnam et al. 2010).

Despite shared inhibitory signalling, NPY receptor subtypes exhibit distinct tissue distribution and functional roles. In neurogenic inflammation, Y1 and Y2 receptors are the principal mediators: Y1 receptors, localised postsynaptically on vascular smooth muscle and sensory neurons, drive vasoconstriction and direct analgesic effects, whereas Y2 receptors function presynaptically to suppress the release of pro‐inflammatory and pronociceptive neuropeptides from sensory terminals (Wahlestedt et al. 1992; Gibbs and Hargreaves 2008; Killough et al. 2010; Rethnam et al. 2010; Basu and Taylor 2024). Although, there is no current evidence of the presence of Y2 receptor in dental pulp tissue, the modulatory effects of NPY on neuroinflammation and hypersensitivity are most plausibly mediated through coordinated Y1–Y2 signalling, underscoring the relevance of receptor‐specific mechanisms in shaping its analgesic and homeostatic potential, which could take place during restorative procedures and dentine hypersensitivity (Table 1).

**TABLE 1:** Differential G protein‐coupled receptor signalling mechanisms of pro‐inflammatory and counter‐regulatory neuropeptides in human dental pulp.

Neuropeptide	Receptor	G protein	Effect on cAMP/second messengers	Functional outcome	References
SP	NK1	Gq	↑ IP3/Ca^2+^	Vasodilation, plasma extravasation, oedema	Teodoro et al. (2013) and Thom et al. (2021)
CGRP	CALCRL/RAMP1	Gs	↑↑↑ cAMP	Potent vasodilation, nociceptor sensitisation	Zhang et al. (2014) and Kee et al. (2018)
NPY	Y1, Y2, Y5	Gi/Go	↓↓ cAMP	Vasoconstriction, analgesia, inhibition of neuropeptide release	Michel et al. (1998), El Karim et al. (2008), Rethnam et al. (2010) and Gibbs and Hargreaves (2008)

*Note:* Substance P (SP) and calcitonin gene‐related peptide (CGRP) are co‐released from capsaicin‐sensitive C‐fibres during antidromic activation and mediate neurogenic inflammation via excitatory G protein pathways. SP binds to neurokinin‐1 (NK1) receptors coupled to Gq proteins, activating phospholipase C‐β to generate inositol 1,4,5‐trisphosphate (IP3) and diacylglycerol (DAG), thereby mobilising intracellular Ca^2+^ and promoting plasma extravasation and oedema. CGRP engages the calcitonin receptor‐like receptor (CALCRL) in complex with receptor activity‐modifying protein 1 (RAMP1), coupled to Gs proteins that potently stimulate adenylate cyclase, elevating cyclic adenosine monophosphate (cAMP) levels up to 10‐fold and inducing robust arteriolar vasodilation and nociceptor sensitisation via transient receptor potential vanilloid 1 (TRPV1) phosphorylation. In contrast, neuropeptide Y (NPY), released from sympathetic terminals, activates Y1, Y2 and Y5 receptors coupled to inhibitory Gi/Go proteins, which suppress adenylate cyclase activity (reducing cAMP) and close presynaptic voltage‐gated Ca^2+^ channels through direct βγ subunit interactions, thereby preventing vesicular release of SP and CGRP and exerting vasoconstrictor and analgesic effects. In the non‐compliant pulpal chamber, this counter‐regulatory NPY system is essential to prevent excessive CGRP‐mediated vasodilation from triggering a pathological cascade of increased intrapulpal pressure, microvascular collapse, and irreversible tissue necrosis. Upregulation of Y1 receptors has been documented in human carious dentine, representing an endogenous compensatory mechanism. Arrows indicate direction of change in second messenger levels.

Abbreviations: Ca^2+^, calcium ion; CALCRL, calcitonin receptor‐like receptor; cAMP, cyclic adenosine monophosphate; CGRP, calcitonin gene‐related peptide; IP3, inositol 1,4,5‐trisphosphate; NK1, neurokinin‐1 receptor; NPY, neuropeptide Y; RAMP1, receptor activity‐modifying protein 1; SP, substance P.

### 4.5 The Central Role of the Y1 Receptor in Pulpal Homeostasis and Pain Modulation

**FIGURE 3:**
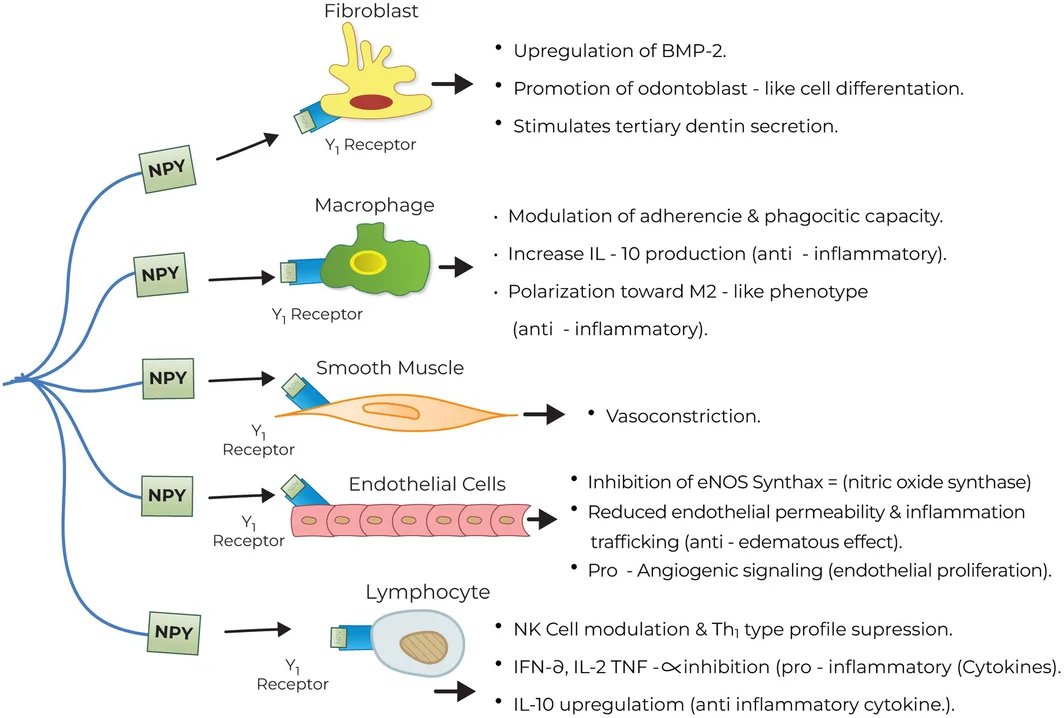
Cell‐specific effects of NPY signalling in the dental pulp. NPY acts as a neuro‐immune ‘Off Switch’. It modulates immunocytes such as macrophages, lymphocytes and NK‐Cells. NPY promotes angiogenesis and tertiary dentine secretion via its effects on fibroblasts and endothelial cells.

NPY restrains neurogenic inflammation in the dental pulp mainly by inhibiting SP release from sensory afferents, thereby limiting amplification of neurogenic inflammatory signalling (El Karim et al. 2008). This effect is mediated predominantly through the Y1 receptor, which is expressed in pulpal vascular smooth muscle cells, fibroblasts, and inflamed odontoblastic–predentine regions—strategic sites where neurogenic inflammation drives vascular dysregulation and nociceptive sensitisation (El Karim, Lamey, Ardill, et al. 2006, El Karim et al. 2008; Killough et al. 2010; Rethnam et al. 2010). In human dental pulp, Y1 is localised along blood vessels in non‐carious tissue and markedly upregulated in nerve fibres, vascular structures, inflammatory cells, and fibroblasts in carious pulps, supporting a protective, suppressive role for NPY Y1 signalling during caries‐induced inflammation and tissue remodelling (El Karim et al. 2008) (Figure 3). Functionally, sympathetic neurotransmitters attenuate SP and CGRP exocytosis from pulpal peptidergic afferents via Y1 activation, reducing inflammatory and nociceptive signalling, while concurrently modulating immune responses through inhibition of T‐cell activation with preservation of antigen‐presenting cell function, with pulpal fibroblasts acting as key neuro‐immune intermediaries (Hargreaves et al. 2003; El Karim, Lamey, Ardill, et al. 2006, 2008; Killough et al. 2010).

Functionally, Y1 receptor activation exerts potent vasoconstrictive and antinociceptive effects that directly oppose the SP‐ and CGRP‐mediated hyperaemia characteristic of early pulpal inflammation. In vascular smooth muscle, NPY Y1 signalling induces vasoconstriction, counterbalancing sensory neuropeptide–driven vasodilation and limiting plasma extravasation and oedema formation (Nilsson et al. 1996; Lundy and Linden 2004; Edwall et al. 1985; Aalkjær et al. 2021). In parallel, Y1 activation suppresses CGRP release from capsaicin‐sensitive, TRPV1‐expressing nociceptors, thereby reducing sensory fibre excitability and inflammatory pain transmission (Gibbs et al. 2004; Gibbs and Hargreaves 2008). Together, these vascular and neural actions define Y1 signalling as a key endogenous mechanism for limiting neurogenic hyperaemia and nociceptive amplification within the dental pulp. It has been proposed that NPY activates G‐protein‐activated inwardly‐rectifying potassium channels and inhibits N‐ and P/Q‐type Ca++ channels, which in turn can inhibit neurotransmitter release from nerve terminals. The Y1 receptor could inhibit nociceptor activity by activating this intracellular pathway. In addition, it is possible that Y1 could activate a phosphatase such as calcineurin, leading to dephosphorylation and desensitisation of TPRV1. NPY has demonstrated peripheral vasopressor effects and immunomodulatory effects that are mediated by Y1 receptors, and it is possible that these could influence the activity of sensory neurons indirectly as well (Gibbs and Hargreaves 2008).

Beyond its vascular actions, Y1 signalling contributes directly to antinociceptive modulation. Y1 receptors are coupled to Gi/Go proteins and inhibit adenylate cyclase, leading to reduced intracellular cAMP levels and downstream modulation of MAPK/ERK signalling pathways, which collectively decrease neuronal excitability (Lecat et al. 2015; Nelson et al. 2024; Michel et al. 1998; Li and Ritter 2005). In the dental pulp, activation of Y1 receptors inhibits capsaicin‐sensitive nociceptors and suppresses CGRP release, providing direct evidence for endogenous antinociceptive control at the peripheral level (Gibbs et al. 2004). This dual capacity to regulate both haemodynamics and sensory signalling positions the Y1 receptor as a central endogenous checkpoint against neurogenic amplification (Gibbs and Hargreaves 2008; Caviedes‐Bucheli, Muñoz, et al. 2008). Collectively, these features identify Y1 as the principal receptor through which NPY may exert clinically meaningful intrinsic analgesic and anti‐inflammatory effects, particularly in the context of dentine hypersensitivity and the mechanical or thermal insults associated with restorative procedures.

### 4.6 Y2 Receptors as Autoregulatory Modulators of Neurogenic Excitation

**FIGURE 4:**
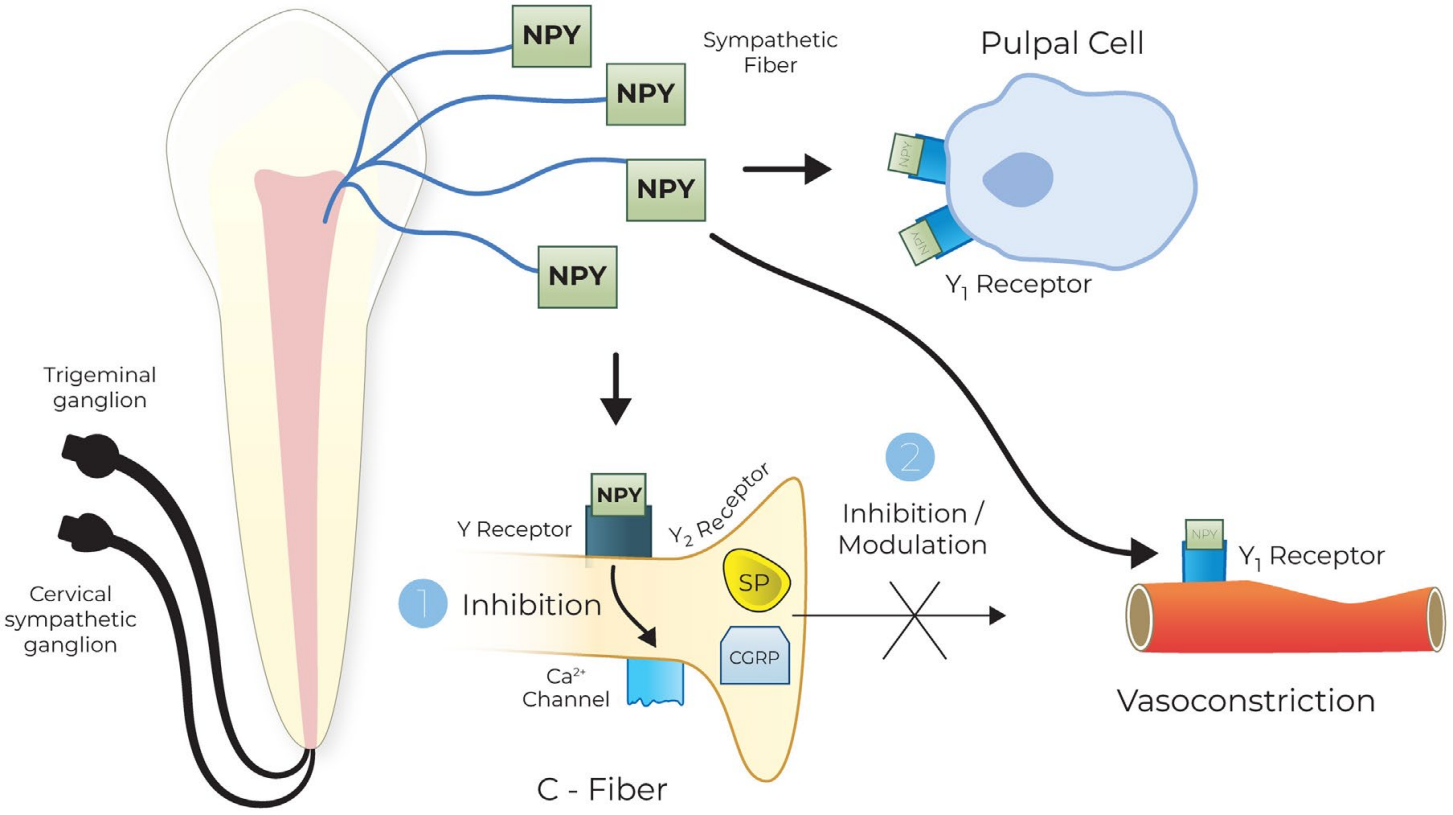
Sympathetic NPY signalling as a counter‐regulatory axis in pulpal neurogenic inflammation, a heterosynaptic inhibition mechanism. Sympathetic sprouting occurs around sensory neurons to counterbalance its pro‐inflammatory effects. NPY release suppresses sensory neuropeptide secretion from C‐fibres through inhibition of Ca^2+^ channels, which in turn reduces vesicular release of SP and CGRP. NPY Y2 signalling functions as a modulatory mechanism in presynaptic terminals by preventing uncontrolled escalation of neurogenic signalling. In contrast, NPY Y1 signalling occurs predominantly post‐synaptically. Y1 expression is upregulated in various types of dental pulp cells.

In some human tissues, Y1 and Y2 receptors constitute a complementary, two‐tiered NPY‐mediated regulatory system. Y1 acts predominantly downstream, stabilising vascular dynamics and suppressing nociceptive excitability, whereas Y2 operates upstream to restrain neuropeptide release and limit excessive neuronal activation (Gerald et al. 1995). This coordinated control might be critical during dental procedures, where rapid and intense sensory inputs are common and where failure of intrinsic inhibitory mechanisms may favour the transition from transient physiological reactivity to sustained neurogenic inflammation and hypersensitivity. Through their distinct anatomical distribution and signalling properties, NPY receptors establish a multi‐layered homeostatic network that counterbalances the potent pro‐inflammatory and hyperalgesic actions of sensory neuropeptides (Parker and Balasubramaniam 2008). Within the unique structural constraints of the dental pulp, this system appears not merely modulatory but essential for preventing progression from reversible irritation to destructive inflammation (Figure 4).

In contrast to the predominantly postsynaptic and vascular actions of Y1, the Y2 receptor functions mainly as a presynaptic auto‐receptor on sympathetic and sensory nerve terminals. Its activation suppresses the release of excitatory neuropeptides—including SP, CGRP, and NPY itself—thereby establishing a negative‐feedback mechanism that limits excessive neuropeptide discharge (Michel et al. 1998; El Karim, Lamey, Linden, et al. 2006; El Karim et al. 2008; Killough et al. 2010). At the molecular level, Y2 signalling engages Gi/Go‐coupled pathways that inhibit adenylate cyclase, reduce intracellular cAMP, and suppress voltage‐gated calcium channel activity, collectively restraining vesicular exocytosis at peripheral nerve endings. This presynaptic inhibitory control is particularly relevant in dentine hypersensitivity, where rapid hydrodynamic stimulation of exposed dentinal tubules triggers abrupt sensory activation of pulpal afferent fibres (Byers and Närhi 1999).

During acute irritative or inflammatory challenges—such as dentinal cutting, thermal fluctuations, osmotic stress, or acidic etching—Y2 receptor engagement functions as an intrinsic braking system that prevents excessive escalation of neurogenic signalling. By attenuating neuropeptide release at presynaptic terminals, Y2 activation limits downstream vasodilatory responses, restrains secondary immune cell recruitment, and dampens peripheral nociceptive amplification (Michel et al. 1998; Killough et al. 2010). In contrast to Y1, Y2 does not directly regulate vascular tone; rather, it acts upstream at the initiation of neurogenic inflammation, fine‐tuning both the magnitude and temporal dynamics of neuropeptide‐driven responses by restricting transmitter release before vascular and immune cascades become fully engaged (Gibbs and Hargreaves 2008; Michel et al. 1998; Parker and Balasubramaniam 2008; Ahmed and Mahdee 2025).

Collectively, this receptor‐mediated framework could provide a mechanistic basis for viewing NPY release during restorative procedures or dentine hypersensitivity as a genuine intrinsic analgesic pathway. By integrating vasoconstriction, modulation of neuronal excitability, and presynaptic control of neuropeptide release, the NPY receptor system functions as a physiological buffer that safeguards pulpal tissue against the consequences of neurogenic over‐activation (Table 2).

**TABLE 2:** Comparative analysis of NPY Y1 and Y2 receptors: Molecular mechanisms, functional roles, and implications in dental pulp nociception and neurogenic inflammation.

Aspect	Y2 receptor (presynaptic)	Y1 receptor (postsynaptic/vascular)	References
Primary localization	Presynaptic nerve terminals, C‐fibres and Aδ‐fibres in dental pulp	Postsynaptic neurons, vascular smooth muscle cells, endothelium, immune cells	Michel et al. (1998), Balasubramaniam et al. (2007) and Blomqvist and Herzog (1997)
G‐Protein coupling	Gi/Go proteins → inhibitory cascade	Gi/Go proteins and Gi/Gq proteins → mixed signalling	Blomqvist and Herzog (1997), Silva et al. (2002) Herzog et al. (1992)
Primary function	Autoreceptor‐mediated negative feedback; limits neuropeptide release	Direct effector receptor; modulates vascular tone, nociception and immune responses	El Karim et al. (2008), Gibbs and Hargreaves (2008) and Parker and Balasubramaniam (2008)
Ion channel modulation	↓ Voltage‐gated Ca^2+^ channels (VGCC); ↑ K^+^ channels → hyperpolarisation	↓ cAMP; modulation of NMDA receptors and voltage‐sensitive Ca^2+^ channels	Acuna‐Goycolea et al. (2005) and Pronchuk et al. (2002)
Second messengers	↓ cAMP/PKA pathway; ↓ intracellular Ca^2+^ (Neurotransmitter inhibition)	↓ cAMP; ↑ intracellular Ca^2+^ (in vascular cells); IP_3_/DAG cascade (Vasoconstriction)	Dumont et al. (1996), Herzog et al. (1992) and Silva et al. (2002)
Effect on neuropeptides	Inhibits release of Substance P (SP), CGRP and NPY itself	Responds to NPY but does not regulate its own release	El Karim et al. (2008), Killough et al. (2010) and Gibbs and Hargreaves (2008)
Vascular effects	Indirect: reduces neurogenic inflammation → ↓ vasodilation, ↓ plasma extravasation	Direct vasoconstriction: contracts smooth muscle; opposes CGRP‐induced vasodilation	Zukowska‐Grojec (1995) and Parker and Balasubramaniam (2008)
Role in nociception	Antinociceptive: dampens peripheral sensitization by limiting excitatory neuropeptide spillover	Mixed: can reduce central pain transmission but also modulate acute nociceptive thresholds	Taiwo and Taylor (2002), Smith et al. (2007) and Gibbs and Hargreaves (2008)
Role in neurogenic inflammation	‘Brake system’: prevents excessive neurogenic inflammation during acute injury (e.g., drilling, thermal stress)	Minimal direct role; primarily modulates vascular responses to inflammation	El Karim et al. (2008) and Zukowska‐Grojec (1995)
Expression in dental pulp	Expressed on sensory nerve fibres; upregulated in inflamed pulp	Present on blood vessels and some nerve fibres; stable expression	Killough et al. (2010), Caviedes‐Bucheli et al. (2005) and El Karim et al. (2008)
Functional plasticity	Expression/function may ↑ during chronic inflammation (compensatory mechanism)	Expression relatively stable; may desensitise under chronic NPY exposure	Ji et al. (1995), Zhang et al. (2006) and Parker and Balasubramaniam (2008)
Therapeutic potential	Y2 agonists: potential to prevent pulpitis escalation and reduce post‐operative pain	Y1 antagonists: potential to reduce vasoconstriction‐related ischemia; improve blood flow	Balasubramaniam et al. (2007), Parker and Balasubramaniam (2008) Silva et al. (2002)
Affinity for NPY	High affinity for NPY and PYY; also binds NPY_3–36_ (truncated form)	High affinity for NPY and NPY_2–36_; low affinity for NPY_3–36_	Michel et al. (1998) Berglund et al. (2003) and Dumont et al. (1998)

*Note:* This table systematically compares the functional, molecular and clinical characteristics of Y1 and Y2 neuropeptide Y (NPY) receptor subtypes in dental pulp neurobiology. Y2 receptors function primarily as presynaptic autoreceptors that inhibit the release of excitatory neuropeptides (substance P, CGRP, NPY), acting as an intrinsic ‘braking system’ during acute inflammatory or irritative challenges. In contrast, Y1 receptors are predominantly postsynaptic and vascular, mediating direct vasoconstriction and modulating nociceptive transmission. Symbols: ↑, increase/upregulation; ↓, decrease/downregulation; →, leads to/results in.

Abbreviations: Ca^2+^, calcium ion; cAMP, cyclic adenosine monophosphate; CGRP, calcitonin gene‐related peptide; DAG, diacylglycerol; DPP‐IV, dipeptidyl peptidase IV; IP_3_, inositol 1,4,5‐trisphosphate; K^+^, potassium ion; NMDA, N‐methyl‐D‐aspartate; NPY, neuropeptide Y; PKA, protein kinase A; PLC, phospholipase C; PYY, peptide YY; SP, substance P; VGCC, voltage‐gated calcium channels.

### 4.7 Clinical Applications of NPY Signalling: Neurogenic Modulation During Restorative and Aesthetic Procedures

Beyond controlled experimental conditions, the dental pulp is continuously exposed to a wide range of clinical noxae—including caries, erosion, occlusal microtrauma, restorative microleakage and operative thermal or mechanical stress—that converge on a common biological pathway: disruption of the dentinal barrier, amplification of dentinal fluid dynamics, and activation of trigeminal sensory afferents. Within this clinically dynamic environment, neuropeptide NPY signalling constitutes a critical modulatory layer that determines whether pulpal responses remain adaptive and reversible or progress towards sustained neurogenic inflammation and irreversible tissue damage (Caviedes‐Bucheli and Munoz 2025).

Restorative procedures provide a clinically relevant model of acute pulpal neurovascular stress. Longitudinal clinical and experimental studies indicate that a measurable proportion of initially vital teeth develop post‐operative pulpal complications following restorative interventions, with reported rates ranging from low single‐digit values to approximately 10%–15% in deep cavities with limited residual dentine (Mjör and Ferrari 2002; Mjör 2009; Duncan 2022). When the residual dentine thickness is more than 2 mm, tissue changes are generally limited to mild subodontoblastic haemorrhage and transient odontoblastic alterations. However, when residual dentine thickness is less than 1 mm, exaggerated plasma extravasation, odontoblastic vacuolisation, and marked disturbances in intratubular fluid dynamics may occur. These changes are accompanied by vasodilation, interstitial oedema, leukocyte recruitment, and microcirculatory stasis, mediated through endothelial adhesion pathways involving ICAM‐1, VCAM‐1, PECAM‐1, selectins and integrins, thereby amplifying neurogenic and inflammatory signalling within the confined pulpal space (Mjör 2009; Caviedes‐Bucheli et al. 2005; Duncan 2022).

At the neurogenic level, caries progression and operative trauma share overlapping molecular signatures. Early dentinal caries increases expression of SP and CGRP within the subodontoblastic plexus, reflecting heightened sensory excitability and a shift towards vasodilatory inflammatory dominance (Byers and Närhi 1999). Similarly, cavity preparation, bleaching procedures, air‐abrasion, and adhesive luting induce bursts of SP and CGRP release, promoting vasodilation, oedema, and immune cell recruitment (Caviedes‐Bucheli et al. 2005, 2021; Caviedes‐Bucheli, Ariza‐García, et al. 2008; Caviedes‐Bucheli and Munoz 2025). When excessive or prolonged, these sensory‐driven responses risk overwhelming pulpal compensatory mechanisms.

Within this reactive environment, NPY provides the principal endogenous counter‐regulatory brake. Released from sympathetic fibres during pulpal stress, NPY acts predominantly through Y1 receptors on vascular smooth muscle cells and fibroblasts to restore vascular tone, limit oedema, and exert direct antinociceptive effects. Concurrently, Y2 autoreceptors on sensory nerve endings suppress further SP and CGRP release, preventing escalation of neurogenic inflammation (Michel et al. 1998; El Karim et al. 2008; Killough et al. 2010; Rethnam et al. 2010). This modulatory capacity is reinforced by the reciprocal structural and functional interplay between sympathetic and sensory nerve networks in the dental pulp. Although NPY‐immunoreactive sympathetic fibres are less numerous than sensory afferents, sympathetic activation can inhibit sensory neuropeptide release and thereby influence reparative processes driven by the somatosensory system. Conversely, selective sympathetic denervation leads to compensatory increases in sensory fibre density, and vice versa, underscoring a tightly regulated homeostatic balance between both neural systems (Haug et al. 2001).

During cavity preparation, sympathetic regulation critically shapes the trajectory of neurogenic pulpal inflammation. Disruption or loss of sympathetic fibres is associated with increased mast cell density, indicating removal of inhibitory control over neurogenic signalling. This loss of sympathetic restraint favours sustained inflammation and heightened pain, partly through increased availability of nerve growth factor (NGF) and unopposed sensory neuropeptide activity (Haug et al. 2001). In the acute phase following cavity preparation, NGF expression is upregulated—primarily by pulpal fibroblasts adjacent to the lesion—driving rapid sensory fibre sprouting and enhanced release of SP and CGRP within the subodontoblastic region. This early sensory hyperinnervation correlates with postoperative pain and dentine hypersensitivity (Ferrari and Byers 1996; Haug et al. 2001).

Beyond its role in nociceptive amplification, NGF also contributes to pulpal repair, as sensory neuropeptide release initiates downstream signalling that supports angiogenesis, resolution of inflammation, and reparative dentinogenesis (Haug et al. 2001). While initial observations suggested limited sprouting of NPY‐immunoreactive sympathetic fibres after cavity preparation—implying differential NGF sensitivity—later evidence indicates that sympathetic sprouting may also occur in an NGF‐dependent manner (Shimeno et al. 2008). A recent article concluded that sensory nerves, but not sympathetic nerves, promote reparative dentine formation. Less reparative dentine was observed beneath the injured dentine of inferior alveolar nerve resected murine molars than in the control group and in the superior cervical ganglion resected molars (Zhan et al. 2024). However, sympathetic denervation has been associated with increased mast cell density and NGF expression, leading to enhanced sensory sprouting and neuropeptide‐mediated inflammation (Haug et al. 2001; Caviedes‐Bucheli et al. 2005). Sympathetic fibres primarily regulate vascular tone through NPY‐mediated mechanisms (Kim and Dörscher‐Kim 1989). Sympathetic stimulation consistently produced vasoconstriction and reduced pulpal blood flow across multiple studies (Tønder and Naess 1978; Kerezoudis, Funato, et al. 1993).

Sympathetic denervation has minimal effects on baseline pulpal hemodynamics but substantially modifies responses to stimulation and injury (Jacobsen and Heyeraas 1997). Chronically sympathectomized animals show 250% larger increases in pulpal blood flow during tooth stimulation compared to intact animals, indicating that sympathetic innervation normally restrains vasodilatory responses (Kerezoudis, Funato, et al. 1993). Beyond vascular effects, sympathetic denervation profoundly affects immune and tissue remodelling processes, increasing immune cell density in dental pulps (Haug et al. 2001), enhancing osteoclast recruitment in periapical lesions (Haug and Heyeraas 2003), increasing root resorption during orthodontic tooth movement (Haug et al. 2003), and altering cytokine expression with increased IL‐1α in inflamed tissues and decreased TNF‐α in intact pulp (Bletsa et al. 2004).

During inflammatory challenges, sympathetic innervation modulated immune cell recruitment differentially. Following electrical tooth stimulation, sympathectomized rats showed no increase in CD43+ cells compared with the unstimulated contralateral control molar, whereas control rats showed significantly more CD43+ PMN cells after stimulation. This suggested that sympathetic nerve stimulation causes recruitment of CD43+ PMN cells (Csillag et al. 2004). These results suggest that under challenging conditions, whether electrical, inflammatory insult, or mechanical stress, sympathetic denervation produced dramatic alterations, but its effects on baseline hemodynamics are minimal (Tønder and Naess 1978). The sympathetic nervous system thus functions primarily as a modulatory system that restrains excessive vascular and inflammatory responses to challenges rather than maintaining baseline homeostasis, with effects mediated through both direct adrenergic and NPY‐mediated vascular control and immune cell regulation (Kerezoudis, Olgart, et al. 1993).

Based on the opposing and complementary effects of sensory and sympathetic innervation in the dental pulp, a regulatory model can be proposed in which postoperative outcomes reflect the dynamic interplay between NGF‐induced sensory activation—driving CGRP‐ and substance P‐mediated inflammation and repair—and NPY‐mediated sympathetic modulation, which regulates vascular tone and limits excessive neurogenic inflammation.

The hydrodynamic characteristics of dentine render this neuromodulatory system particularly relevant. Intratubular fluid shifts—orthodromic or antidromic—generated during operative procedures or by caries‐induced permeability changes are potent activators of mechanosensitive Aδ fibres (Brannstrom 1986; Byers and Närhi 1999). This mechanism underlies dentine hypersensitivity, a clinical phenomenon frequently underappreciated as a neurovascular disorder rather than a purely mechanical one. While the hydrodynamic theory remains the most robust explanatory framework, emerging neuromodulatory perspectives identify NPY as a key regulatory factor. By constraining vasodilation and stabilising microcirculation, NPY indirectly dampens mechanosensitive activation; additionally, through Y1 receptors on nociceptive fibres, it provides a direct analgesic influence.

Adhesive restorative procedures further exemplify the clinical relevance of this balance. Acid conditioning of dentine using agents such as 37% phosphoric acid or itaconic–maleic acid solutions induce dentinal demineralisation, increasing tubule diameter and moisture content to facilitate bonding (Ribeiro et al. 2020). This process triggers immediate vasodilation in the subjacent pulp, likely mediated by SP and CGRP release as part of an intrinsic pulpal defence response (Iványi et al. 2001; Caviedes‐Bucheli, Muñoz, et al. 2008). However, when demineralisation times are prolonged, marked vasoconstriction may occur in the adjacent pulp, probably due to activation of sympathetic NPY‐immunoreactive fibres that compete with sensory fibres for vascular receptors. This shift can result in microcirculatory failure and blood flow stasis, highlighting acid exposure time as a critical determinant of pulpal vascular integrity (Haug et al. 2001; Iványi et al. 2001). Such compromise may precipitate hyperalgesia, neural and vascular injury, and neurogenic inflammatory reactions (Caviedes‐Bucheli, Muñoz, et al. 2008).

Thermal stress generated by light‐curing units represents an additional challenge to pulpal homeostasis. Increases in intrapulpal temperature can provoke inflammatory reactions, with temperature rises of approximately 5.6°C associated with pulpal necrosis in a subset of cases (Kodonas et al. 2009; Savas et al. 2014). In healthy teeth, microcirculatory regulation mediated by sensory, sympathetic, and parasympathetic fibres—through coordinated release of SP, CGRP, NPY and VIP—can mitigate thermal injury (Caviedes‐Bucheli, Muñoz, et al. 2008). Disruption of this balance increases vulnerability to irreversible damage.

Tooth whitening procedures similarly engage neurogenic and neurovascular pathways. Hydrogen peroxide penetration and free radical generation induce cellular injury and release of inflammatory mediators, including prostaglandins, histamine and bradykinin, leading to increased vascular permeability and sensory fibre activation (Cintra et al. 2013; Caviedes‐Bucheli and Munoz 2025). The resulting release of SP and CGRP is accompanied by sympathetic NPY and parasympathetic VIP expression, producing a predominantly reversible inflammatory response characterised clinically by post‐bleaching sensitivity (Caviedes‐Bucheli, Ariza‐García, et al. 2008; Caviedes‐Bucheli, Muñoz, et al. 2008). As inflammation resolves, NPY contributes to restoration of vascular tone and suppression of dentinal hypersensitivity, acting as a natural brake on neurogenic excitation (Caviedes‐Bucheli and Munoz 2025).

Finally, the vasoactive properties of NPY have direct implications for anaesthetic management during restorative procedures. It has been found that 1 min after the infiltration injection of 2% lidocaine with 1:100 000 epinephrine, pulpal blood flow drops off to 60%, and, 6 min later, it reduces to 28%. The recovery of pulpal blood flow may take up to 75 min, and, consequently, during this period of time, dental pulp may experience an inadequate oxygenation (Kim et al. 1984). In healthy pulps, local anaesthetic solutions without vasoconstrictors allow SP‐ and CGRP‐mediated vasodilation, preserving pulpal blood flow (Lin et al. 2009). In contrast, adrenaline‐type vasoconstrictors or felypressin potentiate NPY‐mediated vasoconstriction, markedly reducing pulpal blood flow and increasing pulpal vulnerability. Reduced expression of SP and CGRP under these conditions further diminishes vasodilatory capacity, impairing nutrient delivery and waste removal, and potentially predisposing the pulp–dentine complex to irreversible injury during clinical intervention (Pertl et al. 1997; Caviedes‐Bucheli et al. 2009).

## 5 Knowledge Gaps and Future Directions

Despite compelling evidence positioning NPY as a master regulator of pulpal homeostasis, critical knowledge gaps persist at molecular, cellular, and translational levels.

### 5.1 Molecular Mechanisms

The precise triggering that initiates sympathetic NPY release during pulpal injury remain undefined. Is NPY secretion mediated by depolarization‐dependent exocytosis, ATP‐triggered purinergic signalling, cytokine‐induced activation (IL‐1β, TNF‐α, NGF), or HIF‐1α‐dependent hypoxic pathways? Real‐time microdialysis, optogenetic sympathetic fibre stimulation, and calcium imaging in intact pulp could elucidate NPY release kinetics. Intracellular signalling downstream of Y1/Y2 activation in human pulpal cells—including phosphoproteomic signatures and transcriptional programmes—warrants investigation via single‐cell RNA sequencing and phosphoproteomics.

### 5.2 Regenerative Capacity

NPY's role in odontoblast differentiation and tertiary dentinogenesis remains enigmatic. Does NPY directly promote dental pulp stem cell (DPSC) differentiation? What is the interplay between NPY, Wnt/β‐catenin, and TGF‐β/BMP pathways during reparative dentine formation? Three‐dimensional organotypic cultures and DPSC differentiation assays with selective Y1/Y2 agonists/antagonists are essential.

### 5.3 Therapeutic Translation

No selective Y1 or Y2 receptor agonists have been evaluated in preclinical models of dentine hypersensitivity or post‐operative pain. Development of small‐molecule Y1 agonists for topical application (desensitising varnishes) or Y2 agonists to suppress neurogenic inflammation during high‐risk procedures (deep cavity preparation, bleaching) represents an urgent priority. Proof‐of‐concept human trials assessing topical Y1 agonist efficacy in cervical dentine hypersensitivity or intracanal NPY delivery during vital pulp therapy are critically needed.

### 5.4 Personalised Medicine

Individual variability in NPY expression and receptor polymorphisms may predict pain thresholds and treatment responses. Genome‐wide association studies examining NPY/Y1R/Y2R single nucleotide polymorphisms in dentine hypersensitivity cohorts could identify genetic predictors. Development of chairside salivary/gingival crevicular fluid NPY assays would enable real‐time assessment of endogenous analgesic capacity, guiding treatment decisions.

### 5.5 Biomaterial Integration

Incorporation of NPY or Y1 agonists into bioactive materials (resin‐modified glass ionomers, calcium silicate liners) or nanoparticle delivery systems (PLGA microspheres) could provide sustained neuromodulation during critical healing phases, representing a frontier in biologically informed restorative dentistry.

## 6 Limitations

This review must be interpreted within several methodological constraints:

*Descriptive evidence base*: A substantial proportion of evidence derives from immunohistochemical studies demonstrating NPY/Y receptor spatial expression but not establishing functional causality. Immunoreactivity does not confirm receptor activation or downstream signalling efficacy. Functional validation via receptor binding assays, second messenger quantification, and electrophysiology is essential.

*Limited human in vivo data*: Mechanistic understanding largely derives from animal models (rat, cat) or ex vivo human tissue. Critical species differences exist: rodent continuous eruption versus human dentition; divergent pulpal anatomy, vascular architecture, and receptor expression profiles. Direct translation requires cautious interpretation pending human clinical validation.

*Absence of controlled clinical trials*: No randomised controlled trials have assessed NPY‐based interventions in dentine hypersensitivity or pulpal inflammation. Clinical efficacy, optimal dosing, delivery methods and safety profiles remain unestablished.

*Methodological heterogeneity*: Included studies exhibit variability in tissue processing, antibody specificity, quantification methods, and inflammatory models (bacterial versus mechanical versus chemical stimuli), complicating direct comparisons and meta‐analytical synthesis.

*Temporal dynamics*: Most studies provide static snapshots rather than longitudinal tracking of NPY expression during inflammatory progression. The critical transition points where endogenous NPY regulation becomes insufficient remain undefined.

Despite these limitations, the convergent evidence from multiple methodologies and species supports NPY's central regulatory role, justifying intensified translational research efforts.

## 7 Conclusion

NPY emerges as a central homeostatic regulator of dental pulp neurobiology, integrating vascular control, neurogenic inflammation, and nociceptive modulation through coordinated Y1‐ and Y2‐receptor signalling. Through Y1‐mediated vasoconstriction and direct inhibition of TRPV1‐expressing nociceptors, together with Y2‐dependent presynaptic suppression of substance P and CGRP release, NPY establishes a critical counter‐regulatory axis that restrains sensory neuropeptide–driven inflammation. The balance between excitatory somatosensory signalling and sympathetic NPY‐mediated inhibition ultimately determines whether pulpal injury resolves through adaptive repair or progresses towards irreversible inflammatory degeneration.

Clinically, NPY signalling modulates procedural pain, dentine hypersensitivity, and post‐operative pulpal inflammation across a wide range of restorative and aesthetic interventions. NPY acts as an intrinsic brake on neurogenic overactivation, limiting both vascular dysregulation and nociceptive amplification.

Despite robust experimental evidence, significant translational gaps remain, including incomplete understanding of the molecular triggers of NPY release, intracellular signalling pathways in human pulpal cells, and the lack of controlled clinical trials. Addressing these limitations through advanced molecular, translational, and clinical research will be essential to exploit NPY signalling as a biologically informed therapeutic target.

Repositioning NPY from a secondary compensatory mediator to a potential master regulator of pulpal neurovascular and neuroimmune homeostasis, this work supports a paradigm shift from symptom‐based management towards biologically targeted strategies aimed at preventing postoperative hypersensitivity, preserving pulpal vitality, and optimising outcomes in operative and regenerative endodontics.

## Author Contributions

**Javier Caviedes‐Bucheli:** conceptualisation, formal analysis, investigation, resources, methodology, project administration, supervision, validation, data curation, writing, writing – original draft preparation, writing – review and editing. **Nestor Ríos‐Osorio:** conceptualisation, formal analysis, investigation, resources, methodology, project administration, supervision, validation, data curation, writing, writing – original draft preparation, writing – review and editing. **Esteban Ulate:** investigation, resources, validation, data curation. **Hugo‐Roberto Munoz:** formal analysis, investigation, resources, project administration, supervision, validation, data curation.

## Ethics Statement

The authors have nothing to report.

## Conflicts of Interest

The authors declare no conflicts of interest.

## Data Availability

The authors have nothing to report.

## References

[iej70192-bib-0117] Aalkjær, C. , H. Nilsson , and J. G. R. De Mey . 2021. “Sympathetic and Sensory‐Motor Nerves in Peripheral Small Arteries.” Physiological Reviews 101, no. 2: 495–544. 33270533 10.1152/physrev.00007.2020

[iej70192-bib-0001] Acuna‐Goycolea, C. , N. Tamamaki , Y. Yanagawa , K. Obata , and A. N. van den Pol . 2005. “Mechanisms of Neuropeptide Y, Peptide YY, and Pancreatic Polypeptide Inhibition of Identified Green Fluorescent Protein‐Expressing GABA Neurons in the Hypothalamic Neuroendocrine Arcuate Nucleus.” Journal of Neuroscience 25, no. 32: 7406–7419. 16093392 10.1523/JNEUROSCI.1008-05.2005 PMC6725307

[iej70192-bib-0002] Ahmed, M. S. , and A. F. Mahdee . 2025. “The Role of Neurogenic Inflammation in Pulp Repair and the Techniques Used for Its Assessment (Narrative Review).” Frontiers in Dental Medicine 6: 1686734. 41141899 10.3389/fdmed.2025.1686734 PMC12546036

[iej70192-bib-0003] Aminoshariae, A. , and J. C. Kulild . 2021. “Current Concepts of Dentinal Hypersensitivity.” Journal of Endodontics 47, no. 11: 1696–1702. 34302871 10.1016/j.joen.2021.07.011

[iej70192-bib-0004] Balasubramaniam, A. , R. Joshi , C. Su , L. A. Friend , and J. H. James . 2007. “Neuropeptide Y (NPY) Y2 Receptor‐Selective Agonist Inhibits Food Intake and Promotes Fat Metabolism in Mice: Combined Anorectic Effects of Y2 and Y4 Receptor‐Selective Agonists.” Peptides 28, no. 2: 235–240. 17204349 10.1016/j.peptides.2006.08.041

[iej70192-bib-0005] Basu, P. , and B. K. Taylor . 2024. “Neuropeptide Y Y2 Receptors in Acute and Chronic Pain and Itch.” Neuropeptides 108: 102478. 39461244 10.1016/j.npep.2024.102478 PMC12527949

[iej70192-bib-0006] Berggreen, E. , H. Wiig , and A. Virtej . 2020. “Fluid Transport From the Dental Pulp Revisited.” European Journal of Oral Sciences 128, no. 5: 365–368. 32794278 10.1111/eos.12733

[iej70192-bib-0007] Berglund, M. M. , P. A. Hipskind , and D. R. Gehlert . 2003. “Recent Developments in Our Understanding of the Physiological Role of PP‐Fold Peptide Receptor Subtypes.” Experimental Biology and Medicine 228, no. 3: 217–244. 12626767 10.1177/153537020322800301

[iej70192-bib-0008] Bletsa, A. , K. J. Heyeraas , S. R. Haug , and E. Berggreen . 2004. “IL‐1 Alpha and TNF‐Alpha Expression in Rat Periapical Lesions and Dental Pulp After Unilateral Sympathectomy.” Neuroimmunomodulation 11, no. 6: 376–384. 15467353 10.1159/000080148

[iej70192-bib-0009] Blomqvist, A. G. , and H. Herzog . 1997. “Y‐Receptor Subtypes—How Many More?” Trends in Neurosciences 20, no. 7: 294–298. 9223221 10.1016/s0166-2236(96)01057-0

[iej70192-bib-0010] Brannstrom, M. 1986. “The Hydrodynamic Theory of Dentinal Pain: Sensation in Preparations, Caries, and the Dentinal Crack Syndrome.” Journal of Endodontics 12, no. 10: 453–457. 3465849 10.1016/S0099-2399(86)80198-4

[iej70192-bib-0011] Bryniarska‐Kubiak, N. , A. Basta‐Kaim , and A. Kubiak . 2024. “Mechanobiology of Dental Pulp Cells.” Cells 13, no. 5: 375. 38474339 10.3390/cells13050375 PMC10931140

[iej70192-bib-0012] Byers, M. R. , and M. V. Närhi . 1999. “Dental Injury Models: Experimental Tools for Understanding Neuroinflammatory Interactions and Polymodal Nociceptor Functions.” Critical Reviews in Oral Biology and Medicine 10, no. 1: 4–39. 10759425 10.1177/10454411990100010101

[iej70192-bib-0013] Byers, M. R. , H. Suzuki , and T. Maeda . 2003. “Dental Neuroplasticity, Neuro‐Pulpal Interactions, and Nerve Regeneration.” Microscopy Research and Technique 60, no. 5: 503–515. 12619126 10.1002/jemt.10291

[iej70192-bib-0014] Caterina, M. J. , and D. Julius . 2001. “The Vanilloid Receptor: A Molecular Gateway to the Pain Pathway.” Annual Review of Neuroscience 24: 487–517. 10.1146/annurev.neuro.24.1.487 11283319

[iej70192-bib-0015] Caviedes‐Bucheli, J. , G. Ariza‐García , S. Restrepo‐Méndez , N. Ríos‐Osorio , N. Lombana , and H. R. Muñoz . 2008. “The Effect of Tooth Bleaching on Substance P Expression in Human Dental Pulp.” Journal of Endodontics 34, no. 12: 1462–1465. 19026874 10.1016/j.joen.2008.09.013

[iej70192-bib-0016] Caviedes‐Bucheli, J. , J. A. Correa‐Ortíz , L. V. García , R. López‐Torres , N. Lombana , and H. R. Muñoz . 2005. “The Effect of Cavity Preparation on Substance P Expression in Human Dental Pulp.” Journal of Endodontics 31, no. 12: 857–859. 16306817 10.1097/01.don.0000158237.63383.d8

[iej70192-bib-0017] Caviedes‐Bucheli, J. , N. Lombana , M. M. Azuero‐Holguín , and H. R. Munoz . 2006. “Quantification of Neuropeptides (Calcitonin Gene‐Related Peptide, Substance P, Neurokinin A, Neuropeptide Y and Vasoactive Intestinal Polypeptide) Expressed in Healthy and Inflamed Human Dental Pulp.” International Endodontic Journal 39, no. 5: 394–400. 16640639 10.1111/j.1365-2591.2006.01093.x

[iej70192-bib-0018] Caviedes‐Bucheli, J. , L. F. Lopez‐Moncayo , H. D. Muñoz‐Alvear , et al. 2021. “Expression of Substance P, Calcitonin Gene‐Related Peptide and Vascular Endothelial Growth Factor in Human Dental Pulp Under Different Clinical Stimuli.” BMC Oral Health 21, no. 1: 152. 33757513 10.1186/s12903-021-01519-x PMC7988903

[iej70192-bib-0019] Caviedes‐Bucheli, J. , and H. R. Munoz . 2025. “Dental Innervation and Its Responses to Injury.” In Seltzer and Bender's Dental Pulp Revisited, edited by S. B. McClanahan and R. Ordinola Zapata , 3rd ed., 65–97. Quintessence Publishing Co.

[iej70192-bib-0020] Caviedes‐Bucheli, J. , H. R. Muñoz , M. M. Azuero‐Holguín , and E. Ulate . 2008. “Neuropeptides in Dental Pulp: The Silent Protagonists.” Journal of Endodontics 34, no. 7: 773–788. 18570980 10.1016/j.joen.2008.03.010

[iej70192-bib-0021] Caviedes‐Bucheli, J. , P. Rojas , M. Escalona , et al. 2009. “The Effect of Different Vasoconstrictors and Local Anesthetic Solutions on Substance P Expression in Human Dental Pulp.” Journal of Endodontics 35, no. 5: 631–633. 19410073 10.1016/j.joen.2008.12.020

[iej70192-bib-0022] Chung, G. , S. J. Jung , and S. B. Oh . 2013. “Cellular and Molecular Mechanisms of Dental Nociception.” Journal of Dental Research 92, no. 11: 948–955. 23955160 10.1177/0022034513501877

[iej70192-bib-0023] Cintra, L. T. , F. Benetti , A. C. da Silva Facundo , et al. 2013. “The Number of Bleaching Sessions Influences Pulp Tissue Damage in Rat Teeth.” Journal of Endodontics 39, no. 12: 1576–1580. 24238450 10.1016/j.joen.2013.08.007

[iej70192-bib-0024] Csillag, M. , E. Berggreen , I. Fristad , S. R. Haug , A. Bletsa , and K. J. Heyeraas . 2004. “Effect of Electrical Tooth Stimulation on Blood Flow and Immunocompetent Cells in Rat Dental Pulp After Sympathectomy.” Acta Odontologica Scandinavica 62, no. 6: 305–312. 15848973 10.1080/00016350410010045

[iej70192-bib-0025] de Campos Soriani Azevedo, M. , T. P. Garlet , C. F. Francisconi , et al. 2019. “Vasoactive Intestinal Peptide Immunoregulatory Role at the Periapex: Associative and Mechanistic Evidences From Human and Experimental Periapical Lesions.” Journal of Endodontics 45, no. 10: 1228–1236. 31402064 10.1016/j.joen.2019.06.013

[iej70192-bib-0026] Donaldson, L. F. 2006. “Understanding Pulpitis.” Journal of Physiology 573, no. Pt 1: 2–3. 16581853 10.1113/jphysiol.2006.110049 PMC1779693

[iej70192-bib-0027] Dumont, Y. , A. Fournier , S. St‐Pierre , and R. Quirion . 1996. “Autoradiographic Distribution of [^125^I][Leu^31^,Pro^34^]PYY and [^125^I]PYY_3–36_ Binding Sites in the Rat Brain Evaluated With Two Newly Developed Y1 and Y2 Receptor Radioligands.” Synapse (New York, N.Y.) 22, no. 2: 139–158. 8787130 10.1002/(SICI)1098-2396(199602)22:2<139::AID-SYN7>3.0.CO;2-E

[iej70192-bib-0121] Dumont, Y. , D. Jacques , P. Bouchard , and R. Quirion . 1998. “Species Differences in the Expression and Distribution of the Neuropeptide Y Y1, Y2, Y4, and Y5 Receptors in Rodents, Guinea Pig, and Primates Brains.” Journal of Comparative Neurology 402, no. 3: 372–384. 9853905

[iej70192-bib-0028] Duncan, H. F. 2022. “Present Status and Future Directions‐Vital Pulp Treatment and Pulp Preservation Strategies.” International Endodontic Journal 55, no. Suppl 3: 497–511. 35080024 10.1111/iej.13688 PMC9306596

[iej70192-bib-0116] Edwall, B. , B. Gazelius , A. Fazekas , E. Theodorsson‐Norheim , and J. M. Lundberg . 1985. “Neuropeptide Y (NPY) and Sympathetic Control of Blood Flow in Oral Mucosa and Dental Pulp in the Cat.” Acta Physiologica Scandinavica 125, no. 2: 253–264. 2866663 10.1111/j.1748-1716.1985.tb07714.x

[iej70192-bib-0029] El Karim, I. A. , P. J. Lamey , J. Ardill , G. J. Linden , and F. T. Lundy . 2006. “Vasoactive Intestinal Polypeptide (VIP) and VPAC1 Receptor in Adult Human Dental Pulp in Relation to Caries.” Archives of Oral Biology 51, no. 10: 849–855. 16806045 10.1016/j.archoralbio.2006.04.009

[iej70192-bib-0030] El Karim, I. A. , P. J. Lamey , G. J. Linden , L. A. Awawdeh , and F. T. Lundy . 2006. “Caries‐Induced Changes in the Expression of Pulpal Neuropeptide Y.” European Journal of Oral Sciences 114, no. 2: 133–137. 10.1111/j.1600-0722.2006.00343.x 16630305

[iej70192-bib-0031] El Karim, I. A. , P. J. Lamey , G. J. Linden , and F. T. Lundy . 2008. “Neuropeptide Y Y1 Receptor in Human Dental Pulp Cells of Noncarious and Carious Teeth.” International Endodontic Journal 41, no. 10: 850–855. 18699789 10.1111/j.1365-2591.2008.01436.x

[iej70192-bib-0032] El Karim, I. A. , G. J. Linden , C. R. Irwin , and F. T. Lundy . 2009. “Neuropeptides Regulate Expression of Angiogenic Growth Factors in Human Dental Pulp Fibroblasts.” Journal of Endodontics 35, no. 6: 829–833. 19482180 10.1016/j.joen.2009.03.005

[iej70192-bib-0033] El‐Karim, I. , F. T. Lundy , G. J. Linden , and P. J. Lamey . 2003. “Extraction and Radioimmunoassay Quantitation of Neuropeptide Y (NPY) and Vasoactive Intestinal Polypeptide (VIP) From Human Dental Pulp Tissue.” Archives of Oral Biology 48, no. 3: 249–254. 12648563 10.1016/s0003-9969(02)00213-3

[iej70192-bib-0034] European Society of Endodontology , H. F. Duncan , K. M. Galler , et al. 2019. “European Society of Endodontology Position Statement: Management of Deep Caries and the Exposed Pulp.” International Endodontic Journal 52: 923–934. 30664240 10.1111/iej.13080

[iej70192-bib-0035] Ferrari, A. M. , and M. R. Byers . 1996. “Chronic Dexamethasone Treatment and Its Effects on Sensory Neuropeptides, Pulpal Injury Reactions and Reparative Dentin.” Brain Research 723, no. 1–2: 125–134. 8813389 10.1016/0006-8993(96)00231-4

[iej70192-bib-0036] Galler, K. M. , M. Weber , Y. Korkmaz , M. Widbiller , and M. Feuerer . 2021. “Inflammatory Response Mechanisms of the Dentine‐Pulp Complex and the Periapical Tissues.” International Journal of Molecular Sciences 22, no. 3: 1480. 33540711 10.3390/ijms22031480 PMC7867227

[iej70192-bib-0037] Gerald, C. , M. W. Walker , P. J. Vaysse , C. He , T. A. Branchek , and R. L. Weinshank . 1995. “Expression Cloning and Pharmacological Characterization of a Human Hippocampal Neuropeptide Y/Peptide YY Y2 Receptor Subtype.” Journal of Biological Chemistry 270, no. 45: 26758–26761. 7592910 10.1074/jbc.270.45.26758

[iej70192-bib-0115] Gibbs, J. , C. M. Flores , and K. M. Hargreaves . 2004. “Neuropeptide Y Inhibits Capsaicin‐Sensitive Nociceptors via a Y1‐Receptor‐Mediated Mechanism.” Neuroscience 125, no. 3: 703–709. 15099684 10.1016/j.neuroscience.2004.01.044 PMC4516042

[iej70192-bib-0038] Gibbs, J. L. , and K. M. Hargreaves . 2008. “Neuropeptide Y Y1 Receptor Effects on Pulpal Nociceptors.” Journal of Dental Research 87, no. 10: 948–952. 18809749 10.1177/154405910808701008 PMC2576745

[iej70192-bib-0039] Hargreaves, K. M. , D. L. Jackson , and W. R. Bowles . 2003. “Adrenergic Regulation of Capsaicin‐Sensitive Neurons in Dental Pulp.” Journal of Endodontics 29, no. 6: 397–399. 12814223 10.1097/00004770-200306000-00004

[iej70192-bib-0040] Haug, S. R. , E. Berggreen , and K. J. Heyeraas . 2001. “The Effect of Unilateral Sympathectomy and Cavity Preparation on Peptidergic Nerves and Immune Cells in Rat Dental Pulp.” Experimental Neurology 169, no. 1: 182–190. 11312570 10.1006/exnr.2001.7642

[iej70192-bib-0041] Haug, S. R. , P. Brudvik , I. Fristad , and K. J. Heyeraas . 2003. “Sympathectomy Causes Increased Root Resorption After Orthodontic Tooth Movement in Rats: Immunohistochemical Study.” Cell and Tissue Research 313, no. 2: 167–175. 12851810 10.1007/s00441-003-0753-x

[iej70192-bib-0042] Haug, S. R. , and K. J. Heyeraas . 2003. “Effects of Sympathectomy on Experimentally Induced Pulpal Inflammation and Periapical Lesions in Rats.” Neuroscience 120, no. 3: 827–836. 12895522 10.1016/s0306-4522(03)00269-0

[iej70192-bib-0043] Henry, M. A. , and K. M. Hargreaves . 2007. “Peripheral Mechanisms of Odontogenic Pain.” Dental Clinics of North America 51, no. 1: 19–v. 17185058 10.1016/j.cden.2006.09.007

[iej70192-bib-0044] Herzog, H. , Y. J. Hort , H. J. Ball , G. Hayes , J. Shine , and L. A. Selbie . 1992. “Cloned Human Neuropeptide Y Receptor Couples to Two Different Second Messenger Systems.” Proceedings of the National Academy of Sciences of the United States of America 89, no. 13: 5794–5798. 1321422 10.1073/pnas.89.13.5794 PMC402104

[iej70192-bib-0045] Heyeraas, K. J. , and E. Berggreen . 1999. “Interstitial Fluid Pressure in Normal and Inflamed Pulp.” Critical Reviews in Oral Biology and Medicine 10, no. 3: 328–336. 10759412 10.1177/10454411990100030501

[iej70192-bib-0046] Heyeraas, K. J. , and I. Kvinnsland . 1992. “Tissue Pressure and Blood Flow in Pulpal Inflammation.” Proceedings of the Finnish Dental Society 88, no. Suppl 1: 393–401. 1508897

[iej70192-bib-0047] Hossain, M. Z. , M. M. Bakri , F. Yahya , H. Ando , S. Unno , and J. Kitagawa . 2019. “The Role of Transient Receptor Potential (TRP) Channels in the Transduction of Dental Pain.” International Journal of Molecular Sciences 20, no. 3: 526. 30691193 10.3390/ijms20030526 PMC6387147

[iej70192-bib-0048] Hwang, D. D. , S. J. Lee , J. H. Kim , and S. M. Lee . 2021. “The Role of Neuropeptides in Pathogenesis of Dry Eye.” Journal of Clinical Medicine 10, no. 18: 4248. 34575359 10.3390/jcm10184248 PMC8471988

[iej70192-bib-0049] Iványi, I. , B. Kispélyi , A. Fazekas , L. Rosivall , and I. Nyárasdy . 2001. “The Effect of Acid Etching on Vascular Diameter of Pulp‐Vessels in Rat Incisor (Vitalmicroscopic Study).” Operative Dentistry 26, no. 3: 248–252. 11357566

[iej70192-bib-0050] Jacobsen, E. B. , and K. J. Heyeraas . 1997. “Pulp Interstitial Fluid Pressure and Blood Flow After Denervation and Electrical Tooth Stimulation in the Ferret.” Archives of Oral Biology 42, no. 6: 407–415. 9382705 10.1016/s0003-9969(97)00037-x

[iej70192-bib-0051] Ji, R. R. , Q. Zhang , P. Y. Law , H. H. Low , R. Elde , and T. Hökfelt . 1995. “Expression of Mu‐, Delta‐, and Kappa‐Opioid Receptor‐Like Immunoreactivities in Rat Dorsal Root Ganglia After Carrageenan‐Induced Inflammation.” Journal of Neuroscience 15, no. 12: 8156–8166. 8613750 10.1523/JNEUROSCI.15-12-08156.1995 PMC6577939

[iej70192-bib-0052] Jiang, W. , S. Sun , D. Wang , et al. 2022. “MicroRNA‐22 Suppresses NLRP3/CASP1 Inflammasome Pathway‐Mediated Proinflammatory Cytokine Production by Targeting the HIF‐1α and NLRP3 in Human Dental Pulp Fibroblasts.” International Endodontic Journal 55, no. 11: 1225–1240. 35979583 10.1111/iej.13814 PMC9805103

[iej70192-bib-0053] Kawashima, N. , and T. Okiji . 2024. “Characteristics of Inflammatory Mediators in Dental Pulp Inflammation and the Potential for Their Control.” Frontiers in Dental Medicine 5: 1426887. 39917698 10.3389/fdmed.2024.1426887 PMC11797954

[iej70192-bib-0054] Kee, Z. , X. Kodji , and S. D. Brain . 2018. “The Role of Calcitonin Gene Related Peptide (CGRP) in Neurogenic Vasodilation and Its Cardioprotective Effects.” Frontiers in Physiology 9: 1249. 30283343 10.3389/fphys.2018.01249 PMC6156372

[iej70192-bib-0055] Kerezoudis, N. P. , A. Funato , L. Edwall , and L. Olgart . 1993. “Activation of Sympathetic Nerves Exerts an Inhibitory Influence on Afferent Nerve‐Induced Vasodilation Unrelated to Vasoconstriction in Rat Dental Pulp.” Acta Physiologica Scandinavica 147, no. 1: 27–35. 8095767 10.1111/j.1748-1716.1993.tb09469.x

[iej70192-bib-0056] Kerezoudis, N. P. , L. Olgart , A. Funato , and L. Edwall . 1993. “Inhibitory Influence of Sympathetic Nerves on Afferent Nerve‐Induced Extravasation in the Rat Incisor Pulp Upon Direct Electrical Stimulation of the Tooth.” Archives of Oral Biology 38, no. 6: 483–490. 8393653 10.1016/0003-9969(93)90184-n

[iej70192-bib-0057] Killough, S. A. , F. T. Lundy , and C. R. Irwin . 2010. “Dental Pulp Fibroblasts Express Neuropeptide Y Y1 Receptor but Not Neuropeptide Y.” International Endodontic Journal 43, no. 10: 835–842. 20636351 10.1111/j.1365-2591.2010.01741.x

[iej70192-bib-0058] Kim, S. 1990. “Neurovascular Interactions in the Dental Pulp in Health and Inflammation.” Journal of Endodontics 16, no. 2: 48–53. 2201743 10.1016/S0099-2399(06)81563-3

[iej70192-bib-0059] Kim, S. , and J. Dörscher‐Kim . 1989. “Hemodynamic Regulation of the Dental Pulp in a Low Compliance Environment.” Journal of Endodontics 15, no. 9: 404–408. 2637333 10.1016/S0099-2399(89)80172-4

[iej70192-bib-0060] Kim, S. , L. Edwall , H. Trowbridge , and S. Chien . 1984. “Effects of Local Anesthetics on Pulpal Blood Flow in Dogs.” Journal of Dental Research 63, no. 5: 650–652. 6584468 10.1177/00220345840630050801

[iej70192-bib-0061] Kodonas, K. , C. Gogos , and D. Tziafas . 2009. “Effect of Simulated Pulpal Microcirculation on Intrapulpal Temperature Changes Following Application of Heat on Tooth Surfaces.” International Endodontic Journal 42, no. 3: 247–252. 19228215 10.1111/j.1365-2591.2008.01508.x

[iej70192-bib-0062] Korkmaz, Y. , M. Plomann , B. Puladi , A. Demirbas , W. Bloch , and J. Deschner . 2023. “Dental Pulp Inflammation Initiates the Occurrence of Mast Cells Expressing the α1 and β1 Subunits of Soluble Guanylyl Cyclase.” International Journal of Molecular Sciences 24, no. 2: 901. 36674416 10.3390/ijms24020901 PMC9861465

[iej70192-bib-0063] Lai, J. , F. Porreca , J. C. Hunter , and M. S. Gold . 2004. “Voltage‐Gated Sodium Channels and Hyperalgesia.” Annual Review of Pharmacology and Toxicology 44: 371–397. 10.1146/annurev.pharmtox.44.101802.121627 14744251

[iej70192-bib-0064] Lambiase, A. , A. Micera , M. Sacchetti , M. Cortes , F. Mantelli , and S. Bonini . 2011. “Alterations of Tear Neuromediators in Dry Eye Disease.” Archives of Ophthalmology 129, no. 8: 981–986. 21825181 10.1001/archophthalmol.2011.200

[iej70192-bib-0065] Larhammar, D. , and E. Salaneck . 2004. “Molecular Evolution of NPY Receptor Subtypes.” Neuropeptides 38, no. 4: 141–151. 15337367 10.1016/j.npep.2004.06.002

[iej70192-bib-0124] Lecat, S. , L. Belemnaba , J. L. Galzi , and B. Bucher . 2015. “Neuropeptide Y Receptor Mediates Activation of ERK1/2 via Transactivation of the IGF Receptor.” Cellular Signalling 27, no. 7: 1297–1304. 25817573 10.1016/j.cellsig.2015.03.016

[iej70192-bib-0066] Lee, K. , B. M. Lee , C. K. Park , Y. H. Kim , and G. Chung . 2019. “Ion Channels Involved in Tooth Pain.” International Journal of Molecular Sciences 20, no. 9: 2266. 31071917 10.3390/ijms20092266 PMC6539952

[iej70192-bib-0126] Li, A. J. , and S. Ritter . 2005. “Functional Expression of Neuropeptide Y Receptors in Human Neuroblastoma Cells.” Regulatory Peptides 129, no. 1‐3: 119–124. 15927706 10.1016/j.regpep.2005.02.003

[iej70192-bib-0067] Lin, C. T. , H. Y. Wang , Y. J. Tsai , C. T. Huang , S. H. Chen , and J. H. Lue . 2009. “Pre‐Treatment With Lidocaine Suppresses Ectopic Discharges and Attenuates Neuropeptide Y and c‐Fos Expressions in the Rat Cuneate Nucleus Following Median Nerve Transection.” Journal of Chemical Neuroanatomy 38, no. 1: 47–56. 19559985 10.1016/j.jchemneu.2009.03.003

[iej70192-bib-0068] Lin, Y. T. , and J. C. Chen . 2018. “Dorsal Root Ganglia Isolation and Primary Culture to Study Neurotransmitter Release.” Journal of Visualized Experiments 140: 57569. 10.3791/57569 PMC6235431 30346383

[iej70192-bib-0069] Lundy, F. T. , and G. J. Linden . 2004. “Neuropeptides and Neurogenic Mechanisms in Oral and Periodontal Inflammation.” Critical Reviews in Oral Biology and Medicine 15, no. 2: 82–98. 15059944 10.1177/154411130401500203

[iej70192-bib-0070] Luo, W. , M. Yang , C. Zhan , et al. 2026. “Unveiling the Vital Role of Dental Nerves in Dental Pulp Immune Defence and Repair.” International Endodontic Journal 59, no. 1: 2–18. 41045001 10.1111/iej.70040

[iej70192-bib-0071] Michel, M. C. , A. Beck‐Sickinger , H. Cox , et al. 1998. “XVI. International Union of Pharmacology Recommendations for the Nomenclature of Neuropeptide Y, Peptide YY, and Pancreatic Polypeptide Receptors.” Pharmacological Reviews 50, no. 1: 143–150. 9549761

[iej70192-bib-0072] Mjör, I. A. 2009. “Dentin Permeability: The Basis for Understanding Pulp Reactions and Adhesive Technology.” Brazilian Dental Journal 20, no. 1: 3–16. 19466224 10.1590/s0103-64402009000100001

[iej70192-bib-0073] Mjör, I. A. , and M. Ferrari . 2002. “Pulp‐Dentin Biology in Restorative Dentistry. Part 6: Reactions to Restorative Materials, Tooth‐Restoration Interfaces, and Adhesive Techniques.” Quintessence International (Berlin, Germany: 1985) 33, no. 1: 35–63. 11887534

[iej70192-bib-0074] Mjör, I. A. , and D. Odont . 2001. “Pulp‐Dentin Biology in Restorative Dentistry. Part 2: Initial Reactions to Preparation of Teeth for Restorative Procedures.” Quintessence International (Berlin, Germany: 1985) 32, no. 7: 537–551. 11495566

[iej70192-bib-0075] Moore, E. R. , B. Michot , O. Erdogan , A. Ba , J. L. Gibbs , and Y. Yang . 2022. “CGRP and Shh Mediate the Dental Pulp Cell Response to Neuron Stimulation.” Journal of Dental Research 101, no. 9: 1119–1126. 35403480 10.1177/00220345221086858 PMC9305843

[iej70192-bib-0076] Neiva, K. G. , H. I. J. Coe , D. Peinado , et al. 2015. “Substance P and Neuropeptide Y Expression in Dental Pulp Following Traumatic Injuries.” International Journal of Dentistry and Oral Sciences 2, no. 7: 97–101.

[iej70192-bib-0125] Nelson, T. S. , H. N. Allen , and R. Khanna . 2024. “Neuropeptide Y and Pain: Insights From Brain Research.” ACS Pharmacology & Translational Science 7, no. 12: 3718–3728. 39698268 10.1021/acsptsci.4c00333 PMC11651174

[iej70192-bib-0077] Nie, A. A. , and B. K. Taylor . 2025. “The Pharmacotherapeutic Potential of Neuropeptide Y for Chronic Pain.” Journal of Internal Medicine 298, no. 4: 280–296. 40754889 10.1111/joim.20118 PMC12459321

[iej70192-bib-0114] Nilsson, T. , L. Cantera , and L. Edvinsson . 1996. “Presence of Neuropeptide Y Y1 Receptor Mediating Vasoconstriction in Human Cerebral Arteries.” Neuroscience Letters 204, no. 3: 145–148. 8938251 10.1016/0304-3940(96)12315-6

[iej70192-bib-0078] Olgart, L. 1996. “Neural Control of Pulpal Blood Flow.” Critical Reviews in Oral Biology and Medicine 7, no. 2: 159–171. 8875030 10.1177/10454411960070020401

[iej70192-bib-0079] Page, M. J. , J. E. McKenzie , P. M. Bossuyt , et al. 2021. “The PRISMA 2020 Statement: An Updated Guideline for Reporting Systematic Reviews.” BMJ (Clinical Research Ed.) 372: n71. 10.1136/bmj.n71 PMC8005924 33782057

[iej70192-bib-0080] Parker, S. L. , and A. Balasubramaniam . 2008. “Neuropeptide Y Y2 Receptor in Health and Disease.” British Journal of Pharmacology 153, no. 3: 420–431. 17828288 10.1038/sj.bjp.0707445 PMC2241788

[iej70192-bib-0081] Pertl, C. , R. Amann , E. Odell , P. D. Robinson , and S. Kim . 1997. “Effects of Local Anesthesia on Substance P and CGRP Content of the Human Dental Pulp.” Journal of Endodontics 23, no. 7: 416–418. 9587292 10.1016/S0099-2399(97)80293-2

[iej70192-bib-0082] Pohl, S. , T. Akamp , M. Smeda , et al. 2024. “Understanding Dental Pulp Inflammation: From Signaling to Structure.” Frontiers in Immunology 15: 1474466. 39534600 10.3389/fimmu.2024.1474466 PMC11554472

[iej70192-bib-0083] Pozo, D. , M. Delgado , M. Martínez , et al. 2000. “Immunobiology of Vasoactive Intestinal Peptide (VIP).” Immunology Today 21, no. 1: 7–11. 10637552 10.1016/s0167-5699(99)01525-x

[iej70192-bib-0084] Pronchuk, N. , A. G. Beck‐Sickinger , and W. F. Colmers . 2002. “Multiple NPY Receptors Inhibit GABA(A) Synaptic Responses of Rat Medial Parvocellular Effector Neurons in the Hypothalamic Paraventricular Nucleus.” Endocrinology 143, no. 2: 535–543. 11796508 10.1210/endo.143.2.8655

[iej70192-bib-0085] Renton, T. , Y. Yiangou , P. A. Baecker , A. P. Ford , and P. Anand . 2003. “Capsaicin Receptor VR1 and ATP Purinoceptor P2X3 in Painful and Nonpainful Human Tooth Pulp.” Journal of Orofacial Pain 17, no. 3: 245–250. 14520770

[iej70192-bib-0086] Rethnam, S. , B. Raju , I. Fristad , E. Berggreen , and K. J. Heyeraas . 2010. “Differential Expression of Neuropeptide Y Y1 Receptors During Pulpal Inflammation.” International Endodontic Journal 43, no. 6: 492–498. 20536577 10.1111/j.1365-2591.2010.01704.x

[iej70192-bib-0087] Ribeiro, A. P. D. , N. T. Sacono , D. G. Soares , E. A. F. Bordini , C. A. de Souza Costa , and J. Hebling . 2020. “Human Pulp Response to Conventional and Resin‐Modified Glass Ionomer Cements Applied in Very Deep Cavities.” Clinical Oral Investigations 24, no. 5: 1739–1748. 31372829 10.1007/s00784-019-03035-3

[iej70192-bib-0088] Rodd, H. D. , A. R. Loescher , and F. M. Boissonade . 1998. “Immunocytochemical and Electron‐Microscopic Features of Tooth Pulp Innervation in Hereditary Sensory and Autonomic Neuropathy.” Archives of Oral Biology 43, no. 6: 445–454. 9717582 10.1016/s0003-9969(98)00025-9

[iej70192-bib-0089] Ronan, E. A. , M. Nagel , and J. J. Emrick . 2024. “The Anatomy, Neurophysiology, and Cellular Mechanisms of Intradental Sensation.” Frontiers in Pain Research 5: 1376564. 38590718 10.3389/fpain.2024.1376564 PMC11000636

[iej70192-bib-0090] Russell, F. A. , R. King , S. J. Smillie , X. Kodji , and S. D. Brain . 2014. “Calcitonin Gene‐Related Peptide: Physiology and Pathophysiology.” Physiological Reviews 94, no. 4: 1099–1142. 25287861 10.1152/physrev.00034.2013 PMC4187032

[iej70192-bib-0091] Savas, S. , M. S. Botsali , E. Kucukyilmaz , and T. Sari . 2014. “Evaluation of Temperature Changes in the Pulp Chamber During Polymerization of Light‐Cured Pulp‐Capping Materials by Using a VALO LED Light Curing Unit at Different Curing Distances.” Dental Materials Journal 33, no. 6: 764–769. 25311340 10.4012/dmj.2013-274

[iej70192-bib-0092] Shiau, H. J. 2012. “Dentin Hypersensitivity.” Journal of Evidence‐Based Dental Practice 12, no. 3 Suppl: 220–228. 10.1016/S1532-3382(12)70043-X 23040350

[iej70192-bib-0093] Shimeno, Y. , Y. Sugawara , M. Iikubo , N. Shoji , and T. Sasano . 2008. “Sympathetic Nerve Fibers Sprout Into Rat Odontoblast Layer, but Not Into Dentinal Tubules, in Response to Cavity Preparation.” Neuroscience Letters 435, no. 1: 73–77. 18342446 10.1016/j.neulet.2008.02.015

[iej70192-bib-0094] Silva, A. P. , C. Cavadas , and E. Grouzmann . 2002. “Neuropeptide Y and Its Receptors as Potential Therapeutic Drug Targets.” Clinica Chimica Acta; International Journal of Clinical Chemistry 326, no. 1–2: 3–25. 12417094 10.1016/s0009-8981(02)00301-7

[iej70192-bib-0095] Smith, P. A. , T. D. Moran , F. Abdulla , K. K. Tumber , and B. K. Taylor . 2007. “Spinal Mechanisms of NPY Analgesia.” Peptides 28, no. 2: 464–474. 17194506 10.1016/j.peptides.2006.09.029

[iej70192-bib-0096] Sorkin, L. S. , K. A. Eddinger , S. A. Woller , and T. L. Yaksh . 2018. “Origins of Antidromic Activity in Sensory Afferent Fibers and Neurogenic Inflammation.” Seminars in Immunopathology 40, no. 3: 237–247. 29423889 10.1007/s00281-017-0669-2 PMC7879713

[iej70192-bib-0097] Szczot, M. , A. R. Nickolls , R. M. Lam , and A. T. Chesler . 2021. “The Form and Function of PIEZO2.” Annual Review of Biochemistry 90: 507–534. 10.1146/annurev-biochem-081720-023244 PMC8794004 34153212

[iej70192-bib-0098] Taiwo, O. B. , and B. K. Taylor . 2002. “Antihyperalgesic Effects of Intrathecal Neuropeptide Y During Inflammation Are Mediated by Y1 Receptors.” Pain 96, no. 3: 353–363. 11973010 10.1016/S0304-3959(01)00481-X

[iej70192-bib-0099] Tan, Y. V. , and J. A. Waschek . 2011. “Targeting VIP and PACAP Receptor Signalling: New Therapeutic Strategies in Multiple Sclerosis.” ASN Neuro 3, no. 4: e00065. 21895607 10.1042/AN20110024 PMC3189630

[iej70192-bib-0100] Tanaka, M. , S. Yamada , and Y. Watanabe . 2021. “The Role of Neuropeptide Y in the Nucleus Accumbens.” International Journal of Molecular Sciences 22, no. 14: 7287. 34298907 10.3390/ijms22147287 PMC8307209

[iej70192-bib-0101] Teodoro, F. C. , M. F. Tronco Júnior , A. R. Zampronio , A. C. Martini , G. A. Rae , and J. G. Chichorro . 2013. “Peripheral Substance P and Neurokinin‐1 Receptors Have a Role in Inflammatory and Neuropathic Orofacial Pain Models.” Neuropeptides 47, no. 3: 199–206. 23177733 10.1016/j.npep.2012.10.005

[iej70192-bib-0102] Thom, C. , J. Ehrenmann , S. Vacca , et al. 2021. “Structures of Neurokinin 1 Receptor in Complex With Gq and Gs Proteins Reveal Substance P Binding Mode and Unique Activation Features. *Science* .” Advances 7, no. 50: eabk2872. 10.1126/sciadv.abk2872 PMC8654284 34878828

[iej70192-bib-0103] Tønder, K. H. , and G. Naess . 1978. “Nervous Control of Blood Flow in the Dental Pulp in Dogs.” Acta Physiologica Scandinavica 104, no. 1: 13–23. 696351 10.1111/j.1748-1716.1978.tb06246.x

[iej70192-bib-0104] Uddman, R. , T. Grunditz , and F. Sundler . 1984. “Neuropeptide Y: Occurrence and Distribution in Dental Pulps.” Acta Odontologica Scandinavica 42, no. 6: 361–365. 6397958 10.3109/00016358409033616

[iej70192-bib-0105] Uddman, R. , J. Kato , L. Cantera , and L. Edvinsson . 1998. “Localization of Neuropeptide Y Y1 Receptor mRNA in Human Tooth Pulp.” Archives of Oral Biology 43, no. 5: 389–394. 9681114 10.1016/s0003-9969(97)00117-9

[iej70192-bib-0106] Wahlestedt, C. , S. Regunathan , and D. J. Reis . 1992. “Identification of Cultured Cells Selectively Expressing Y1‐, Y2‐, or Y3‐Type Receptors for Neuropeptide Y/Peptide YY.” Life Sciences 50, no. 4: PL7–PL12. 1310131 10.1016/0024-3205(92)90342-m

[iej70192-bib-0122] Wakisaka, S. 1990. “Neuropeptides in the Dental Pulp: Distribution, Origins, and Correlation.” Journal of Endodontics 16, no. 2: 67–69. 2388020 10.1016/S0099-2399(06)81566-9

[iej70192-bib-0107] Wakisaka, S. , S. H. Youn , J. Kato , M. Takemura , and K. Kurisu . 1996. “Neuropeptide Y‐Immunoreactive Primary Afferents in the Dental Pulp and Periodontal Ligament Following Nerve Injury to the Inferior Alveolar Nerve in the Rat.” Brain Research 712, no. 1: 11–18. 8705292 10.1016/0006-8993(95)01421-7

[iej70192-bib-0108] Wang, D. , M. Wang , S. Sun , et al. 2024. “Hypoxia‐Induced NLRP3 Inflammasome Activation via the HIF‐1α/NF‐κB Signaling Pathway in Human Dental Pulp Fibroblasts.” BMC Oral Health 24, no. 1: 1156. 39343901 10.1186/s12903-024-04936-w PMC11441079

[iej70192-bib-0109] Yang, C. , Q. Gao , N. Xu , K. Yang , and Z. Bian . 2023. “Human Dental Pulp Stem Cells Are Subjected to Metabolic Reprogramming and Repressed Proliferation and Migration by the Sympathetic Nervous System via α1B‐Adrenergic Receptor.” Journal of Endodontics 49, no. 12: 1641–1651.e6. 37769871 10.1016/j.joen.2023.09.007

[iej70192-bib-0110] Yu, F. H. , V. Yarov‐Yarovoy , G. A. Gutman , and W. A. Catterall . 2005. “Overview of Molecular Relationships in the Voltage‐Gated Ion Channel Superfamily.” Pharmacological Reviews 57, no. 4: 387–395. 16382097 10.1124/pr.57.4.13

[iej70192-bib-0111] Zhan, C. , M. Huang , J. Chen , Y. Lu , X. Yang , and J. Hou . 2024. “Sensory Nerves, but Not Sympathetic Nerves, Promote Reparative Dentine Formation After Dentine Injury via CGRP‐Mediated Angiogenesis: An In Vivo Study.” International Endodontic Journal 57, no. 1: 37–49. 37874659 10.1111/iej.13989

[iej70192-bib-0112] Zhan, C. , M. Huang , X. Yang , and J. Hou . 2021. “Dental Nerves: A Neglected Mediator of Pulpitis.” International Endodontic Journal 54, no. 1: 85–99. 32880979 10.1111/iej.13400

[iej70192-bib-0118] Zhang, H. , E. W. Roubos , B. G. Jenks , and W. J. Scheenen . 2006. “Receptors for Neuropeptide Y, Gamma‐Aminobutyric Acid and Dopamine Differentially Regulate Ca2+ Currents in Xenopus Melanotrope Cells via the G(i) Protein Beta/Gamma‐Subunit.” General and Comparative Endocrinology 145, no. 2: 140–147. 16214143 10.1016/j.ygcen.2005.08.006

[iej70192-bib-0120] Zukowska‐Grojec, Z. 1995. “Neuropeptide Y. A Novel Sympathetic Stress Hormone and More.” Annals of the New York Academy of Sciences 771: 219–233. 8597401 10.1111/j.1749-6632.1995.tb44683.x

[iej70192-bib-0113] Zukowska‐Grojec, Z. , E. Karwatowska‐Prokopczuk , W. Rose , et al. 1998. “Neuropeptide Y: A Novel Angiogenic Factor From the Sympathetic Nerves and Endothelium.” Circulation Research 83, no. 2: 187–195. 9686758 10.1161/01.res.83.2.187

